# Dissociation Time, Quantum Yield, and Dynamic Reaction Pathways in the Thermolysis of *trans*-3,4-Dimethyl-1,2-dioxetane

**DOI:** 10.1021/acs.jpclett.3c03578

**Published:** 2024-02-09

**Authors:** Jian-Ge Zhou, Yinan Shu, Yuchen Wang, Jerzy Leszczynski, Oleg Prezhdo

**Affiliations:** + Interdisciplinary Nanotoxicity Center, Department of Chemistry, Physics and Atmospheric Sciences, Jackson State University, Jackson, Mississippi 39217, United States; ‡ Department of Chemistry and Supercomputing Institute, University of Minnesota, Minneapolis, Minnesota 55455-0431, United States; & Department of Chemistry and James Franck Institute, The University of Chicago, Chicago, Illinois 60637, United States; $ Department of Chemistry and Department of Physics and Astronomy, University of Southern California, Los Angeles, California 90089, United States

## Abstract

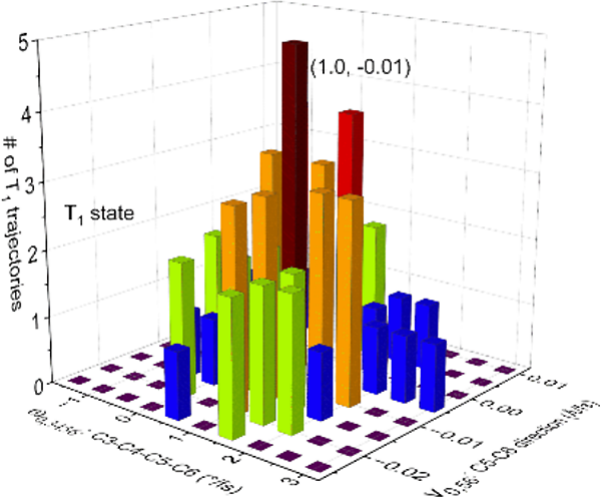

The thermolysis of *trans*-3,4-dimethyl-1,2-dioxetane is studied by trajectory surface hopping. The significant difference between long and short dissociation times is rationalized by frustrated dissociations and the time spent in triplet states. If the C–C bond breaks through an excited state channel, then the trajectory passes over a ridge of the potential energy surface of that state. The calculated triplet quantum yields match the experimental results. The dissociation half-times and quantum yields follow the same ascending order as per the product states, justifying the conjecture that the longer dissociation time leads to a higher quantum yield, proposed in the context of the methylation effect. The populations of the molecular Coulomb Hamiltonian and diagonal states reach equilibrium, but the triplet populations with different S_*z*_ components fluctuate indefinitely. Certain initial velocities, leading the trajectories to given product states, can be identified as the most characteristic features for sorting trajectories according to their product states.

Chemiluminescence represents the emanation of light in a chemical reaction in which a molecule is thermally activated and experiences a nonadiabatic transition to an electronic excited state of the fragmentation product.^1−3^ The process is called bioluminescence when it occurs in living organisms, e.g., fireflies. Chemiluminescence or bioluminescence has wide-ranging applications, such as treating cancers via photodynamic therapy,^4−6^ acting as a bioluminescence imaging tool,^7,8^ and tracking environmental pollutants.^9^ The thermolysis of the four-membered heterocyclic peroxides, e.g., 1,2-dioxetane, generates electronically excited state carbonyl compounds that emit light to release the excess energy^1^ (see Figure 1). The enhancement of the quantum yields of singlet and triplet chemiexcitation was achieved experimentally by the methylation of the 1,2-dioxetanes.^1,10^ The chemiluminescent mechanism has been studied by computing potential energy surface (PESs), locating minima, transition states (TSs), and conical interactions and/or intersystem crossings, and identifying the potential reaction pathways.^11−18^ The dynamical quantities, e.g., dissociation time and branching ratios, however, cannot be analyzed in the context of critical points and PESs. The high demand for the nonadiabatic molecular dynamics (NAMD) methods, in which the nuclear motion is regulated by multiple electronic states, has led to the development of different approaches.^19−33^ As the full quantum-mechanical methods are expensive and limited to a few degrees of freedom,^33−35^ the trajectory surface hopping (TSH)^19^ method has emerged as one of the most popular tools for the NAMD simulations, which was used recently to study the chemiluminescent processes for the four-membered heterocyclic peroxides.^1^

**Figure 1:**
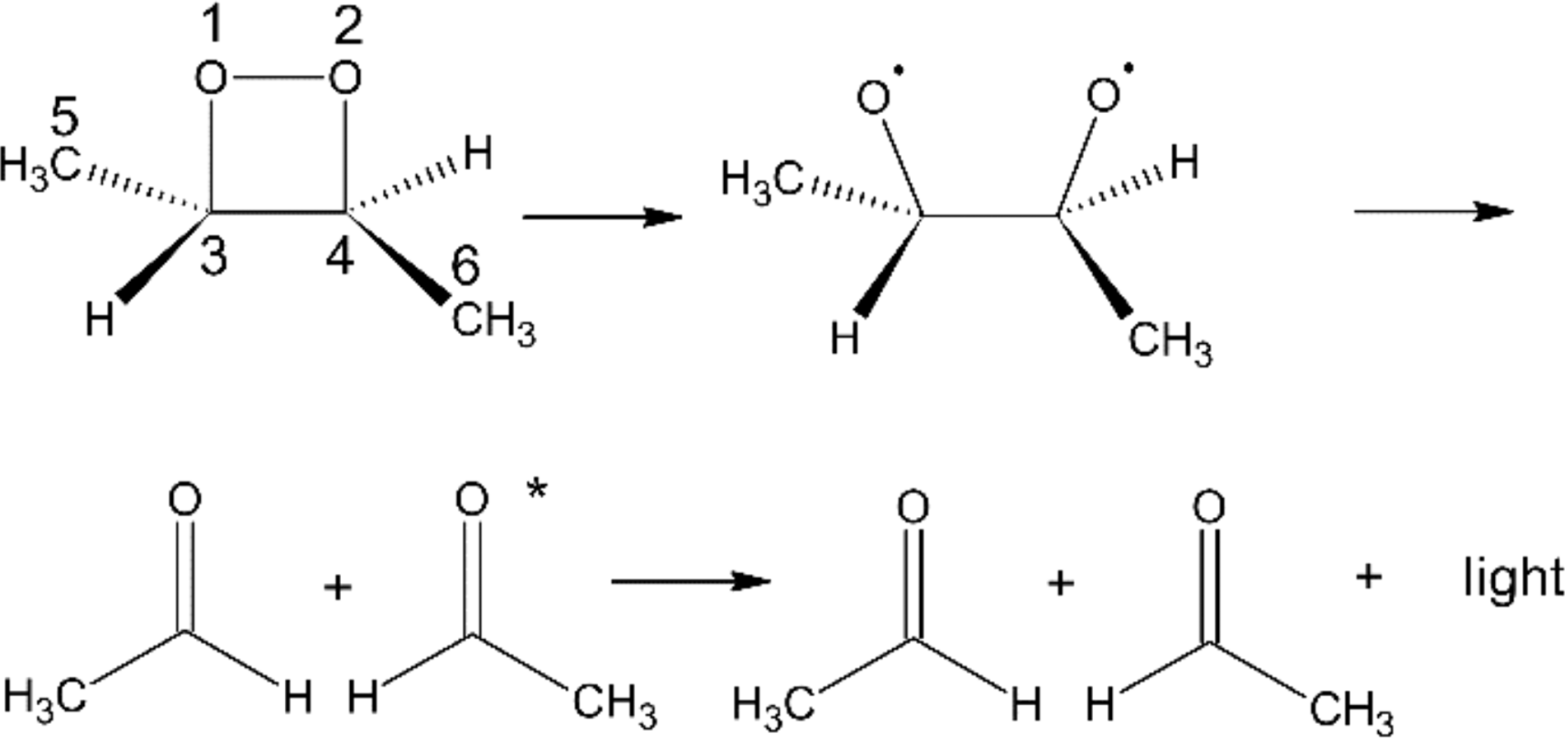
Thermolysis of *trans*-3,4-dimethyl-1,2-dioxetane into two acetaldehydes. The asterisk means that the molecule is in the excited state.

The thermal decomposition of 1,2-dioxetane derivatives was discussed via the TSH approach^36,37^ implemented in the Newton-X package^38^ interfaced with Molcas^39^ (Newton-X/Molcas). The electronic structure method employed in Molcas^39^ was the state-averaged complete active space self-consistent field (SA-CASSCF)^40^ with the ANO-RCC-VTZP basis set^41^ and state averaging over the four lowest singlet states in the active space of 12 electrons and 10 orbitals, i.e., SA4-CASSCF(12e,10o)/ANO-RCC-VTZP. It inferred that the 1,2-dioxetane molecule is trapped in the singlet excited states, and the decomposition process is deferred.^36^ The methyl substitution of the 1,2-dioxetane increased the dissociation time via the mass effect in which the dissociation time was reproduced by the substitution groups with the same moment of inertia of the methyl group.^37^ With the Newton-X/Molcas combined package, the quantum yield of *trans*-3,4-dimethyl-1,2-dioxetane (TDMD)^10^ was estimated by the SA8-CASSCF(8e,6o)/STO-3G+6-31G* mixed basis set, including the four lowest singlet states and four lowest triplet states.^42^ The obtained data revealed that the calculated quantum yield of the first triplet excited state (T_1_) was comparable to the experimental result,^10^ and the quantum yield of the second singlet excited state (S_2_) was greater than that of the first singlet excited state (S_1_). Including the triplet excited states in the TSH simulation increases the dissociation half-time enormously, and this is the reason that the small active space and mixed basis set were selected.^42^ Even with these achievements, some questions are still unclear. (1) As the fragmentation product of the trajectories, i.e., the acetaldehyde molecule (Figure 1), can be in different energy states (e.g., T_1_, S_0_, or S_1_), what is the order of the dissociation times as per the product energy states, or what state has the longest dissociation half-time? (2) Why do some trajectories have short dissociation times, some long, and is it related to the initial conditions of trajectories? (3) Why is the quantum yield of the S_2_ product state greater than that of the S_1_ product state, and what mechanism is behind this anomaly? (4) After the molecule escapes the entropic trap region^12,36^ in which the four lowest singlet states and four lowest triplet states are nearly degenerated, do the surface hops still take place, and can the populations of the triplet states with different *z* components of the spin reach the constant values after a certain time period? (5) Are there some traits that make it most obvious for them to assort the trajectories according to their product energy states?

Guided by these questions, in this work we study the dynamic thermolysis of the TDMD molecule by the TSH method^19^ implemented in the SHARC package^43−45^ interfaced with OpenMolcas.^46^ One of the strengths of the SHARC is that it processes internal conversion and intersystem crossing on the same footing by combining the spin–orbit-free input energies, gradients, and nonadiabatic couplings with the spin–orbit matrix elements in a spin–orbit-free electronic basis. As the decomposition of the TDMD can be characterized by the O1–O2–C3–C4 dihedral angle (Figure 1), we evaluate the distribution of the trajectories over this dissociation dihedral angle and dissociation time, minimum and maximum dissociation times, and dissociation half-time to demonstrate the allocation of the trajectories, and the order of the dissociation times as per their product energy states. The time evolutions of the C3–C4 distance, the O1–O2–C3–C4 dihedral angle, the potential energy of the active state, the number of frustrated dissociations, and the time spans in the singlet and triplet states along the short and long trajectories are calculated to explain the significant dissociation time difference between the short and long trajectories and address the cause of delay of the decomposition process. We compute the time evolutions of the ensemble-averaged populations of the MCH (molecular Coulomb Hamiltonian) and diagonal states^44^ to examine the quantum yields for the respective product energy states and check the population equilibrium of the triplet excited states with different *z* components of the spin. To explore how the product energy state of the trajectories stochastically correlates with the initial velocities, we evaluate the distribution of the number of the trajectories over the initial velocities and dissociation time to determine if in the initial velocities there are the most characteristic features that are easiest to use to separate the trajectories as per their product energy states.^47^

For the evaluation of electronic structure of the biradicals in the thermolysis of the TDMD, the SA-CASSCF approach^40^ with state averaging over states S_0_–S_3_ and T_1_–T_4_ was applied to optimize the transition states, evaluate the normal modes at the transition states, and compute the potential energy and its first derivative with respect to nuclear coordinates along the trajectories. The selected active space consists of the 12 electrons distributed in 10 orbitals, i.e., the four σ and four σ* orbitals of the four-membered ring and the two oxygen lone-pair orbitals perpendicular to the ring.^36,37^ The 6-31G basis set^48^ was employed in the optimization and NAMD simulation, i.e., SA8-CASSCF(12e,10o)/6-31G. The dynamic process starting from the O–O transition state (Figure 1 and Figure S1) can be simulated by the SA-CASSCF method (see Figure S2).

We conducted the NAMD simulations by the fewest-switches trajectory surface hopping^19^ with energy-based decoherence correction^49−51^ implemented in SHARC 2.1.^43−45^ The nuclear equations of motion were integrated by the velocity-Verlet algorithm^52^ with a time step size of 0.5 fs and propagated by using the projected nonadiabatic couplings as the direction to adjust the momentum after the trajectory hops.^53^ The electronic structure is handled quantum mechanically by solving the time-independent Schrödinger equation, and the nuclei propagate in trajectories governed by multiple potential energy surfaces. The total Hamiltonian for intersystem crossing dynamics in the SHARC is written as *H*^total^ = *H*^MCH^ + *H*^SOC^, where *H*^MCH^ is the nonrelativistic molecular Coulomb Hamiltonian (MCH) and the *H*^SOC^ is the spin–orbit coupling operator.^54^ The eigenstates of *H*^MCH^ are denoted as the MCH states, e.g., states S_0_ and T_1,–1_, in which the total electron spin *S* and its *z* component S_z_ are good quantum numbers. If the spin–orbit couplings (SOCs) are included,^54^ there are two drawbacks in the MCH representation. (1) The off-diagonal couplings in *H*^total^ are usually delocalized over the PES, which results in the non-zero transition probability even far from crossing regions; thus, a much larger number of trajectories are needed to sample the process correctly because surface hops may occur in a much larger phase space volume.^44^ (2) The sum of the transition probabilities over all multiple components depends on the rotation of the molecule in the laboratory frame.^55^ To overcome these shortcomings, the diagonal basis is selected, which is the set of eigenstates of *H*^total^, i.e., diagonal states.^44^ All couplings between the diagonal states are described by a nonadiabatic coupling vector. To simulate the nonadiabatic dynamics of the thermal decomposition of the TDMD effectively, all sampling trajectories start from the rate-controlling O–O bond-breaking transition state (TS_O–O_) in the S_0_ state (Figure 1 and Figure S1) and have the same initial coordinates of TS_O–O_, but the initial velocities are generated by the random sampling algorithm proposed by Sellner, Barbatti, and Lischka (SBL).^56^ The initial kinetic energy at TS_O–O_ (3.96 eV) is the zero-point vibrational energy of TS_O–O_ (3.35 eV, computed via SA-CASSCF) plus the CASPT2 correction energy (see the Supporting Information). First, the initial velocities are sampled by the Boltzmann distribution at 300 K, and then the initial kinetic energies of all of the trajectories at TS_O–O_ are rescaled to the same energy (3.96 eV). In the O–O reaction coordinate with the imaginary frequency, the sampled velocities, forward to the product direction, are selected for the NAMD simulation. We have coded the SBL sampling algorithm in SHARC. The C–C van der Waals bond length is 2.4 Å, and the trajectories are forced to be terminated if the distance between the C3 and C4 atoms is >3.7 Å. The electronic energies, nuclear gradients, and SOCs are all computed using OpenMolcas 21.06^46^ that is interfaced with SHARC version 2.1.^43−45^

In the NAMD simulation, we consider the S_0_–S_3_ and T_1_–T_4_ states among which the surface hopping to state T_3_ or T_4_ is turned off to reduce the computational cost and simulation time. Of the 310 trajectories, 302 trajectories terminated successfully with a C3–C4 distance of >3.7 Å, and eight trajectories failed because of the lack of conservation of energy. All trajectories end in the T_1_, S_0_, and S_1_ product state, and no trajectory was found in the other states. The MCH state of the trajectory labeled at the time of the cleavage of the C3–C4 bond (it just becomes greater than 2.4 Å) remains unchanged after the C3–C4 bond breaks. For example, if the MCH state of the trajectory is T_1_ around 2.4 Å of the C3–C4 distance, the trajectory follows the T_1_ state until it reaches 3.7 Å (the *S*_*z*_ components still change). The distribution of the number of the completed trajectories over the dissociation time (the time needed when the C–C distance evolves to 2.4 Å) and O1–O2–C3–C4 dissociation dihedral angle in Figure 2 shows that (1) the maximum dissociation times for the T_1_, S_0_, and S_1_ trajectories, which have corresponding T_1_, S_0_, and S_1_ product state, are ∼1600, ∼4500, and ∼1200 fs respectively; (2) the most prevalent dissociation dihedral angles are almost the same for the T_1_, S_0_, and S_1_ trajectories and are around 150.0° (the initial dihedral angle is 135.8°; the most populated angle variation between the final and initial dihedral angle of the trajectories is 14.2°) and the most populated trajectories have short dissociation times; (3) the O1–O2–C3–C4 dissociation dihedral angle of the trajectories is distributed from −180° to 0° to 180° (−180° is equal to 180° in a dihedral angle); and (4) most S_1_ trajectories have short dissociation times.

**Figure 2:**
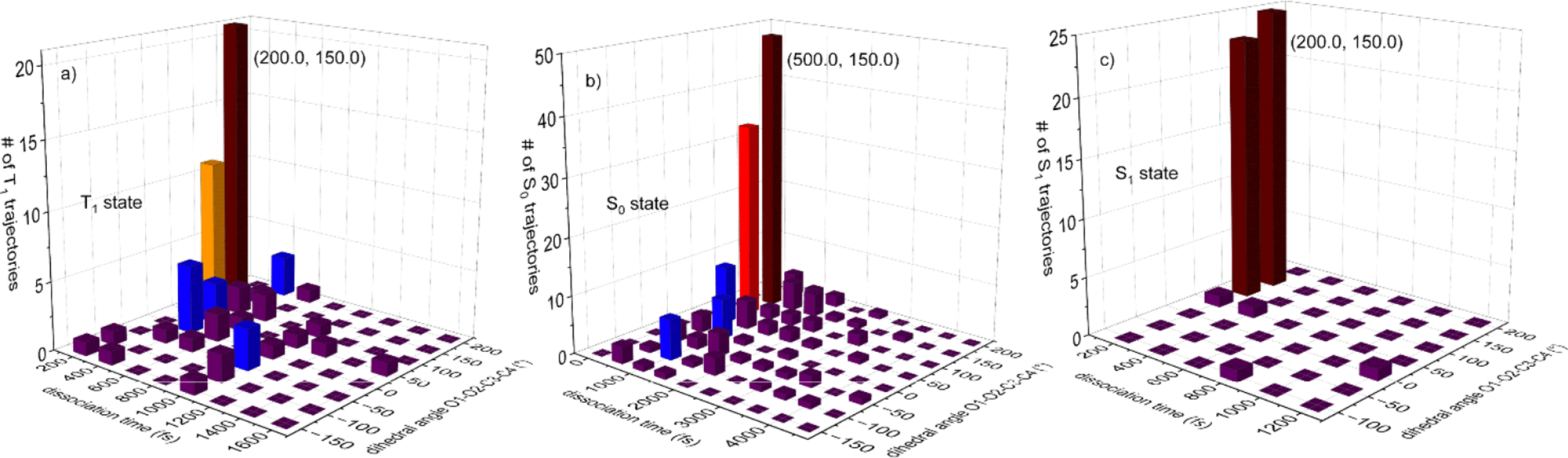
Distribution of the number of trajectories over the dissociation time (femtoseconds) and O1–O2–C3–C4 dissociation dihedral angle (degrees) for the (a) T_1_, (b) S_0_, and (c) S_1_ product states.

The minimum and maximum dissociation times, dissociation half-times, means, and standard deviations are listed in Table 1 for the T_1_, S_0_, and S_1_ product states. The dissociation half-time is the time required for half of the trajectories to dissociate. Table 1 indicates that the S_0_ trajectories have the longest dissociation half-time of 253.25 fs, and the S_1_ trajectories have the shortest dissociation half-time of 64.50 fs. Both orders of the dissociation half-times and maximum dissociation times are the same for the T_1_, S_0_, and S_1_ product states. The maximum dissociation time perhaps provides the time scale for the chemiluminescent decomposition. For the S_0_ trajectories, the shortest trajectory takes only 39.00 fs for dissociation to occur, but the longest one needs 4329.00 fs to break the C3–C4 bond. The dissociation time difference between the long and short S_0_ trajectories is significant, and similar conclusions hold for the T_1_ and S_1_ trajectories.

**Table 1:** Minimum and Maximum Dissociation Times, Dissociation Half-Times, Means, and Standard Deviations (femtoseconds) for the T_1_, S_0_, and S_1_ Product States

	T_1_	S_0_	S_1_	all trajectories
minimum dissociation time	46.50	39.00	44.50	39.00
maximum dissociation time	1566.50	4329.00	1155.00	4329.00
dissociation half-time	196.00	253.25	64.50	178.00
mean	343.82	667.01	111.43	496.44
standard deviation	320.65	860.21	176.80	720.08

To see how the short and long trajectories behave differently in their dissociation times, we study the time evolutions of the C3–C4 distance and the O1–O2–C3–C4 dihedral angle (Figure 3a,b). For the T_1_ short trajectory (trajectory 69, a representative of the T_1_ short trajectories) whose product state is T_1_, the C3–C4 distance increases almost monotonously with time; however, the dihedral angle changes from 135.8° to 128.9°, and the angle variation is 6.9°, which demonstrates that this T_1_ short trajectory dissociates without any frustrated dissociation.^36^ At 44.0 fs, the T_1_ short trajectory is on the PES of the T_1_ state, its potential energy reaches the maximum [3.27 eV (Figure 3c)], and the corresponding C3–C4 distance is 2.16 Å (Figure 3a), which is greater than 2.08 Å, which is the C3–C4 distance of the C–C transition state on the T_1_ PES (Figure S1c). At 42.5 fs, the dihedral angle arrives at its local maximum. Before 42.5 fs, the dihedral angle increases, and at 42.5 fs, the dihedral angle begins to decrease to prepare the configuration to pass over the ridge of the T_1_ PES between TS_O–O_ and the dissociated TDMD, which is termed the T_1_ ridge (the crossing point over this T_1_ ridge is the potential energy peak at 44.0 fs). Most peaks (e.g., at 18.5 fs) along the potential energy curve of the active state represent the surface hops between the diagonal states, in which the dihedral angles at the corresponding times of the potential energy peaks do not display the locally minimal or maximal values. The nonadiabatic transitions between the MCH states in this T_1_ short trajectory are listed in Table 2. The diagonal element represents the number of transitions within a MCH state (step *i* to step *i* + 1 within a MCH state is counted as one transition), and the off-diagonal element shows the number of surface hops between two different MCH states. The total steps in a MCH state are the diagonal element of this state plus the number of the surface hops from all other states to this state, which is the sum of all of the off-diagonal elements along the column in which this state resides (Table 2) (see the Supporting Information for details).

**Figure 3:**
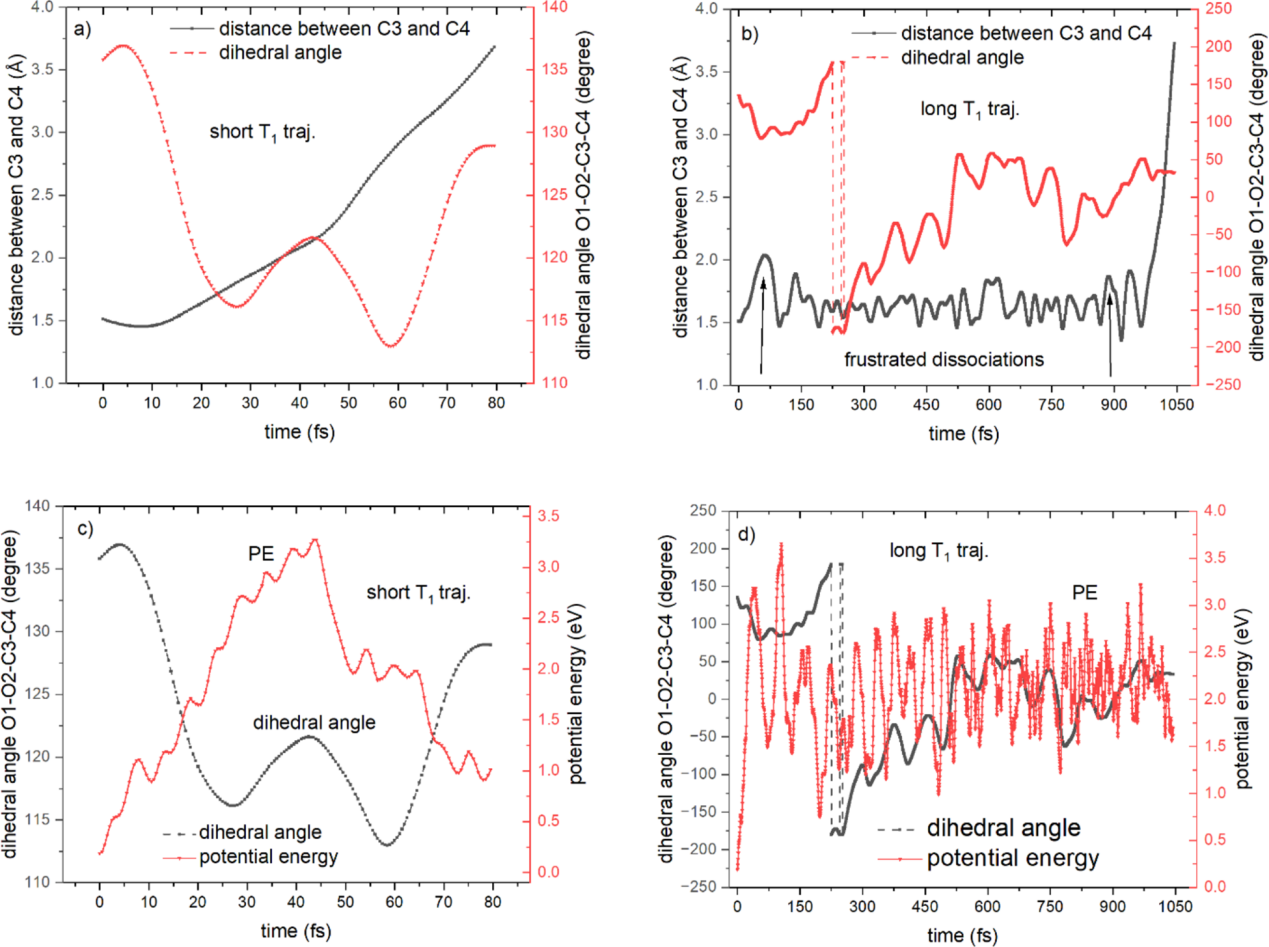
Time evolutions of the C3–C4 distance (angstroms) and O1–O2–C3–C4 dihedral angle (degrees) for (a) a T_1_ short trajectory and (b) a T_1_ long trajectory. The peaks along the C3–C4 curve represent the frustrated dissociations, and the vertical arrows indicate two frustrated dissociations. Time evolution of the O1–O2–C3–C4 dihedral angle and the potential energy (electronvolts) of the active state for (c) a T_1_ short trajectory and (d) a T_1_ long trajectory.

**Table 2:** Numbers of Nonadiabatic Transitions among the MCH States in the T_1_ Short Trajectory

	S_0_	S_1_	S_2_	S_3_	T_1,–1_	T_2,–1_	T_1,0_	T_2,0_	T_1,1_	T_2,1_
S_0_	59	0	0	0	1	0	0	0	0	0
S_1_	1	10	0	0	0	0	0	0	0	0
S_2_	0	0	0	0	0	0	0	0	0	0
S_3_	0	0	0	0	0	0	0	0	0	0
T_1,–1_	1	0	0	0	5	0	0	1	0	0
T_2,–1_	0	1	0	0	0	0	0	0	0	0
T_1,0_	0	0	0	0	0	0	0	0	0	0
T_2,0_	0	0	0	0	0	1	0	1	0	0
T_1,1_	0	0	0	0	1	0	0	0	60	1
T_2,1_	0	0	0	0	0	0	0	0	1	15

The C3–C4 bond in this T_1_ short trajectory takes 159 steps, i.e., 79.5 fs (step size of 0.5 fs), to reach 3.7 Å. From Table 2, we find that the T_1_ short trajectory spends 30.5 fs (61 steps), 5.5 fs, 34.0 fs, and 9.5 fs in states S_0_, S_1_, T_1_, and T_2_, respectively, and stays in the T_1_ state with the longest time. This T_1_ short trajectory (1) starts from TS_O–O_ on the S_0_ PES; (2) passes through the entropic trap region by hopping among the S_0_, S_1_, T_1_, and T_2_; and (3) climbs on the T_1_ PES and passes over the T_1_ ridge. In other words, the molecule is dissociated with the T_1_ product state. In general, in the early stage the trajectory is mainly in the S_0_ state; later, it spends its most time in the T_1_ state with the surface hops among the T_1_ states with different *z* components of the spin [*S*_*z*_ = −1, 0, or 1; i.e., T_1,–1_, T_1,0_, and T_1,1_ (the energy gaps among T_1,–1_, T_1,0_, and T_1,1_ are small even out of the entropic trap region), and finally, it overcomes the T_1_ ridge.

For the T_1_ long trajectory (trajectory 23), the dihedral angle changes from 135.8° to 180° (−180°) to −90° to 0° to 32.9° and the angle variation is 257.1°. A dihedral angle takes a value in the ranges of 0° to 180° and 0° to −180° instead of 0° → 180° → 360°; in other words, 180° equals −180°. If an angle oscillates between 178° and 183°, however, for the dihedral angle, it acts as if it is oscillating between 178° and −177°, so it looks discontinuous but continuous geometrically, which explains why the time evolution of the dihedral angle in Figure 3b appears discontinuous between 180° and −180°. The C3–C4 distance oscillates around 1.6 Å, and there are 24 frustrated dissociations within 975.5 fs, which are identified by the local maxima in the time evolution of the C3–C4 bond length [the number of the peaks along the curve of the C3–C4 distance is that of the frustrated dissociations (see Figure 3b)].^36^ The average oscillation period is 40.6 fs (975.5/24) which matches the C–C vibration in the biradical region. In Figure 3d, there are ∼24 noticeable potential energy peaks, which means that each frustrated dissociation can be visualized as the oscillation between the two adjacent local maxima of the potential energy curve. The global maximal potential energy along the T_1_ long trajectory is 3.66 eV located at 105.5 fs; however, it is on the T_2_ PES, and the molecule does not dissociate by overcoming the T_2_ ridge. The T_1_ long trajectory hops among states S_0_–S_3_, T_1_, and T_2_ in the entropic trap region and stochastically seeks its dynamic reaction path. At 966.5 fs, the trajectory is on the T_1_ PES, and the potential energy reaches the second highest peak [3.22 eV (Figure 3d)], which is identified as the other crossing point over the T_1_ ridge. At the same time, the dihedral angle attains its peak (∼966.5 fs), which means that the C3–C4 bond starts to break upon adjustment of the dihedral angle from increasing to decreasing (Figure 3b). After 966.5 fs, the C3–C4 distance rapidly increases to 3.7 Å. From Table 3, we find that the T_1_ long trajectory spends 30.5, 113.5, 24.5, 12.5, 546.0, and 317.0 fs in states S_0_–S_3_, T_1_, and T_2_, respectively. For the most time, the T_1_ long trajectory stays in the T_1_ and T_2_ states and hops among states T_1,–1_, T_1,0_, T_1,1_, T_2,–1_, T_2,0_, and T_2,1_; however, the molecule does not dissociate through the T_2_ ridge, and the C–C bond breaks by passing over the T_1_ ridge.

**Table 3:** Numbers of Nonadiabatic Transitions among the MCH States in the T_1_ Long Trajectory

	S_0_	S_1_	S_2_	S_3_	T_1,–1_	T_2,–1_	T_1,0_	T_2,0_	T_1,1_	T_2,1_
S_0_	51	0	1	0	2	0	5	1	0	0
S_1_	4	203	4	1	2	1	0	8	1	3
S_2_	0	4	42	0	1	0	0	1	0	1
S_3_	0	0	1	24	0	0	0	0	0	0
T_1,–1_	3	1	0	0	474	3	14	1	16	2
T_2,–1_	0	4	0	0	3	176	0	8	1	10
T_1,0_	2	3	0	0	14	0	266	4	16	0
T_2,0_	0	8	0	0	1	7	4	219	0	9
T_1,1_	1	0	0	0	16	1	15	1	239	1
T_2,1_	0	4	1	0	1	14	0	5	1	158

For the S_0_ short trajectory (trajectory 138), at 28.5 fs the potential energy arrives at its global maximum peak [2.86 eV (Figure S4a)], but it is on the S_3_ PES. One femtosecond later (at 29.5 fs), the trajectory jumps to the S_0_ PES and the C3–C4 bond starts to break (there is no local maximum on the S_0_ PES when the C3–C4 distance is between 2.1 and 2.4 Å); i.e., the dissociation does not encounter the S_0_ ridge. From Table S1, we discern that the S_0_ short trajectory spends 60.1%, 21.0%, 1.5%, 15.9%, and 1.5% of the simulation time in states S_0_–S_3_ and T_2_, respectively; in other words, it stays mainly in state S_0_. From Table S2, we find that the S_0_ long trajectory (trajectory 4) spends its most time in states T_1_ and T_2_ (46.0% and 32.3%, respectively) and 10.1%, 7.2%, 2.7%, and 1.7% in states S_0_–S_3_, respectively. Just before 4298.5 fs, the trajectory was wandering in the T_2_ state and then jumps to S_3_ → S_2_ → S_1_ → S_0_ in the short time period, and the molecule dissociates on the S_0_ PES without overcoming any local S_0_ maximum along the potential energy curve (Figures S3b and S4b). The molecule that breaks the C–C bond through the S_0_ PES does not encounter the S_0_ ridge, which is consistent with the reaction coordinate analysis that after TS_O–O_, the C–C bond breaking transition states on the S_1_–S_3_ and T_1_–T_4_ PES exist, but there is no TS_C–C_ on the S_0_ PES.^12,36^ Between 0.0 and 4300.0 fs, there are 108 frustrated dissociations in the C3–C4 bond oscillation (Figure S4b), and the period is 39.8 fs, which is consistent with the C–C vibration. The 108 frustrated dissociations make this trajectory take a long time to dissociate.

At 38.0 fs, the C3–C4 distance is 2.16 Å, and the S_1_ short trajectory (trajectory 88) is in the S_1_ state and reaches its global maximum (3.19 eV) of the potential energy (Figures S3c and S4c). Thus, the molecule dissociates by crossing the S_1_ ridge located at 38.0 fs. From Table S3, we find that the S_1_ short trajectory spends 37.9% of the time in state S_0_, 2.1% in state T_1_, and the highest percentage, 60.0%, in state S_1_ and dissociates along the S_1_ PES. The S_1_ long trajectory (trajectory 253) spends 16.4%, 15.2%, 3.4%, 6.0%, 37.4%, and 21.6% in states S_0_–S_3_, T_1_, and T_2_, respectively (Table S4); in other words, for the most time, it stays in the triplet excited states. At 1147.0 fs, the S_1_ long trajectory is on the S_1_ PES and reaches its local maximum of the potential energy (2.85 eV) and the corresponding the C3–C4 distance is 2.16 Å (Figures S3d and S4d); thus, we infer this is the other crossing point over the S_1_ ridge. After 1147.0 fs, the C3–C4 bond rapidly breaks. Within 1147.0 fs, the C3–C4 bond oscillates 24 times, and the oscillation period is 47.8 fs, which is comparable with that of the C–C bond vibration.

The average total number of hops among all states is 98.9 per trajectory, but the average total number of nonadiabatic transitions among states T_1,–1_, T_1,0_, T_1,1_, T_2,–1_, T_2,0_, and T_2,1_ states is 61.2 per trajectory, which indicates that most hops in trajectories are among the triplet states because of the small energy gaps among states T_1,–1_, T_1,0_, and T_1,1_ (or T_2,–1_ T_2,0_, and T_2,1_). The short trajectories spend a small amount of time in the triplet states, and the C–C bond breaks directly; however, the long trajectories stay in the triplet states for the most time with multiple frustrated dissociations.

The ensemble-average time evolutions of the MCH state populations for states S_0_–S_3_, T_1_, and T_2_ are illustrated in Figure 4a, which shows the population equilibrium after 4200 fs. The equilibrium populations for states T_1_, S_0_, and S_1_ are 23.2%, 59.6%, and 17.2%, respectively, and are zero for other states. The previous research^42^ found that the quantum yield of state S_2_ was greater than that of state S_1_. This anomalous phenomenon is not observed here. One possible reason is the use of the small active space and low-level basis set in the previous work,^42^ which underestimates the transition probability from state S_2_ to lower states. Panels a and b of Figure 4 show that the state S_0_ population (1) decreases from 100% to 29.8% within 95.5 fs, (2) fluctuates between 95.5 and 190.5 fs, and (3) increases between 190.5 and 4200.0 fs and reaches a constant value 59.6% after 4200.0 fs. The S_1_ population increases and arrives at its maximum of 30.1% at 71.0 fs, then decreases to 17.2% at 3979.5 fs, and finally reaches its equilibrium. The state S_2_ and S_3_ populations increase and achieve their maximum values of 10.3% at 48.0 fs and 7.6% at 49.5 fs, respectively, and then go to zero after 2186.5 fs. The T_2_ population increases and reaches its maximum of 18.5% at 132.0 fs and then decreases and vanishes after 3668.0 fs. The T_1_ population increases and reaches its maximum of 34.8% at 365.5 fs, decreases, and reaches its equilibrium 23.2% after 4200.0 fs. States S_0_–S_3_ and T_1_–T_4_ are not degenerated around the TS_O–O_ structure, and the S_0_ state jumps more easily to state S_1_ than to state T_1_, which explains that before 95.5 fs, the S_1_ population grows faster than the T_1_ population. Later, the trajectories enter the entropic trap region, hopping to state T_1_ becomes easier than before, and the trajectories accumulate favorably on the T_1_ PES (see Table 3), which elucidates that the T_1_ population exceeds the S_1_ population after 157.0 fs.

**Figure 4:**
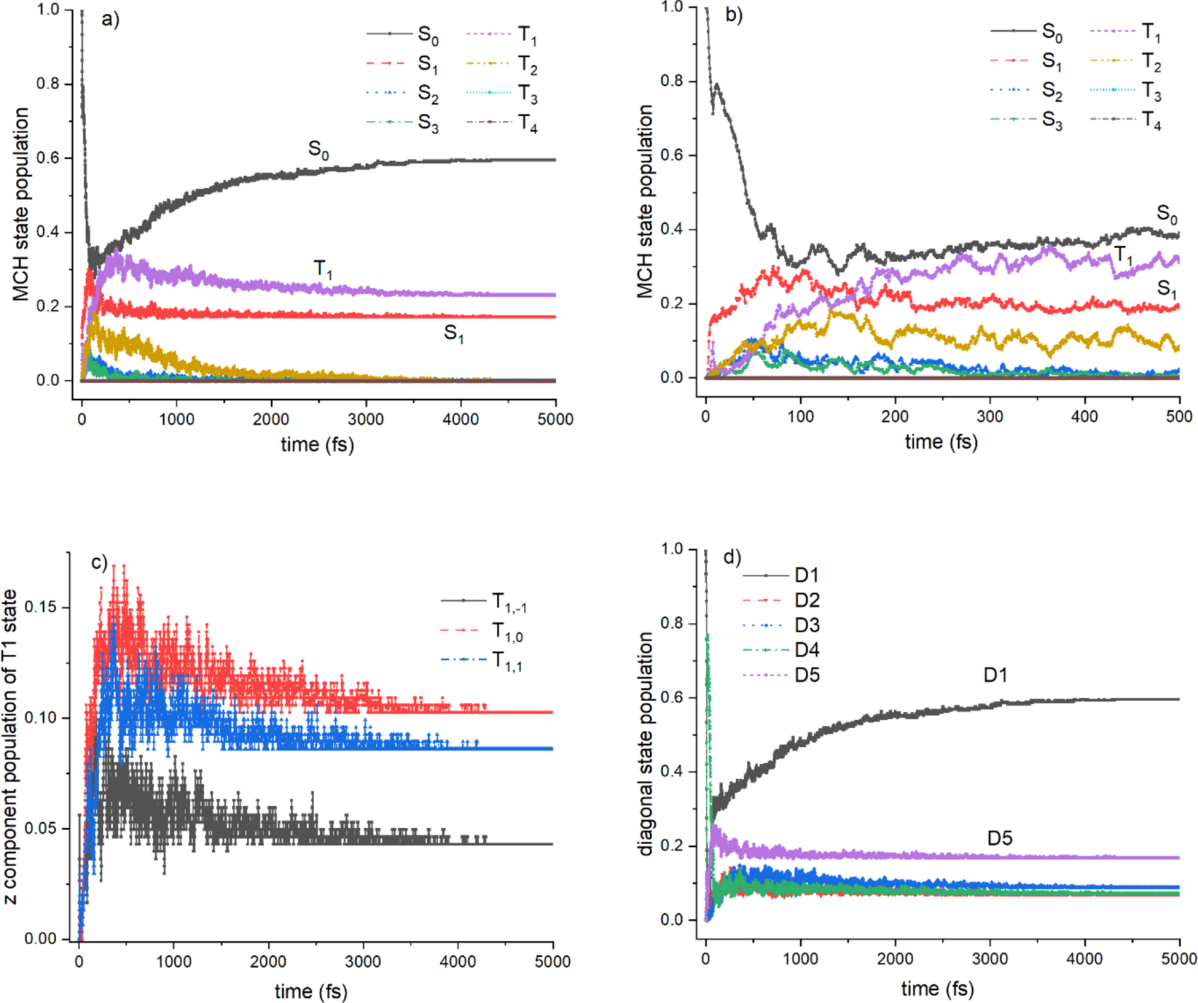
Time evolutions of (a) the populations of the MCH states in 5000 fs, (b) the populations of the MCH states in 500 fs, (c) the populations of states T_1,–1_, T_1,0_, and T_1,1_, and (d) the populations of the diagonal states.

The fragmentation product of the trajectories retains its MCH state (ignoring the difference among triplet states with different *S*_*z*_ values; in other words, T_1,–1_, T_1,0_, and T_1,1_ are termed T_1_) as that labeled at the C–C bond cleavage. If the product is in state S_0_ or S_1_, after scission of the C–C bond, there is no surface hopping in the trajectory. However, if the product is in state T_1_, the surface hops among states T_1,–1_, T_1,0_, and T_1,1_ continue after the C–C bond breaks. Two types of the surface hops occur: (1) between different diagonal states belonging to different MCH states, e.g., second diagonal state (T_1,0_) → third diagonal state (T_1,1_) (the second diagonal state is approximated as the T_1,0_ state in the MCH representation, and the third diagonal state corresponds to the T_1,1_ state), and (2) between different diagonal states belonging to the same MCH state, e.g., fourth diagonal state (T_1,0_) → third diagonal state (T_1,0_). Figure 4c indicates that the populations of states T_1,–1_, T_1,0_, and T_1,1_ fluctuate around 4.3%, 10.3%, and 8.6%, respectively; in other words, they cannot reach population equilibrium because of the small energy differences among them. T_1,0_ is the most populated and the T_1,–1_ the least populated among states T_1,–1_, T_1,0_, and T_1,1_.

Comparing panels a and c of Figure 4 with panel d, we find that the first diagonal state, D1, which is the lowest state in the diagonal representation, mainly contributes to state S_0_ and the major component of state D5 comes from state S_1_. Furthermore, the major parts of states D2–D4 correspond to states T_1,–1_, T_1,0_, and T_1,1_, respectively, but the populations of states D2–D4 can reach equilibrium after 3500 fs. At the TS_O–O_ configuration, state S_0_ has the lowest energy among states S_0_–S_3_ and T_1_–T_4_. Between 6.0 and 15.0 fs, most trajectories (77% of the total number of the trajectories) jump from the first diagonal state (S_0_) to the fourth diagonal state (S_0_) via internal conversion, which implies that state S_0_ evolves from the lowest state (D1) to the fourth lowest state (D4) and the three *z* components of state T_1_ possess energies that are lower than that of state S_0_ in this part of the entropic trap region. This explains why there is a population peak (77.2%) of state D4 (S_0_) around 11.0 fs (see Figure 4d). Here we point out that for most of the time, state D4 corresponds to state T_1,1_ and state D1 matches state S_0_.

The number of trajectories, the quantum yields, and the dissociation half-times for the product MCH states are listed in Table 4. The quantum yields for states S_2_, S_3_, and T_2_ are zero, and the calculated quantum yield of state T_1_ is 23.2%, which matches the experimental value (20.0%). The simulated quantum yield of the singlet excited states (S_1_+S_2_) is 17.2%, which is comparable with the value of 10.1% obtained by the SA8-CASSCF(8e,6o)/STO-3G+6-31G* method.^42^Table 4 shows that for states S_0_, S_1_, and T_1_, the order of the quantum yields is the same as that of the dissociation half-times, which supports the argument that the longer dissociation time should cause the higher quantum yield observed in the mass effect of the methylation.^37^

**Table 4:** Numbers of Trajectories, Quantum Yields, and Dissociation Half-Times (femtoseconds) for the Product Energy States

	S_0_	S_1_	S_2_	S_3_	T_1_	T_2_
number of trajectories	180	52	0	0	70	0
quantum yield (%)	59.6	17.2	0	0	23.2	0
dissociation half-time	253.25	64.50	–	–	196.00	–

To examine whether the product energy states of the dissociated TDMD are related to the initial velocities, first we evaluate the distributions of the S_0_, S_1_, and T_1_ trajectories over the dissociation time and the initial angular velocity of the O1–O2–C3–C4 (or C3–C4–C5–C6) dihedral angle, denoted as ω_0,1234_ (or ω_0,3456_) in Figure 5. The ω_0,1234_ coordinates of the most populated T_1_, S_0_, and S_1_ trajectories are ∼0°/fs (Figure 5a–c); in other words, the highest peaks of product states T_1_, S_0_, and S_1_ cannot be distinguished by ω_0,1234_. The range of ω_0,1234_ for all trajectories is −3.0 ≤ ω_0,1234_ ≤ 2.0; i.e., its range length is 5.0. The subranges are −3.0 ≤ ω_0,1234_ ≤ 1.5, −2.0 ≤ ω_0,1234_ ≤ 2.0, and −2.5 ≤ ω_0,1234_ ≤ 1.0 for the T_1_, S_0_, and S_1_ trajectories, respectively. In the subrange adjacent to the negative end point, −3.0 ≤ ω_0,1234_ < −2.0, whose length (1.0) is 20.0% of the range length (5.0) [this ratio characterizes the relative length of the subrange near an end point (RLSE)], there is no S_0_ trajectory; thus, the trajectories with the ω_0,1234_ close to the negative end point tend to evolve into product states S_1_ and T_1_. In the subrange 1.0 ≤ ω_0,1234_ < 2.0 with a 20.0% RLSE, there is no S_1_ state; thus, the trajectories with the ω_0,1234_ neighboring the positive end develop most likely to states T_1_ and S_0_. From the ω_0,1234_ side view, the number of trajectories on the left side of the highest peak is greater than that on the right side of the highest peak for states T_1_, S_0_, and S_1_, which is attributed to the normal mode along the O1–O2 reaction coordinate being assigned with positive initial velocities to ensure molecular dissociation.

**Figure 5:**
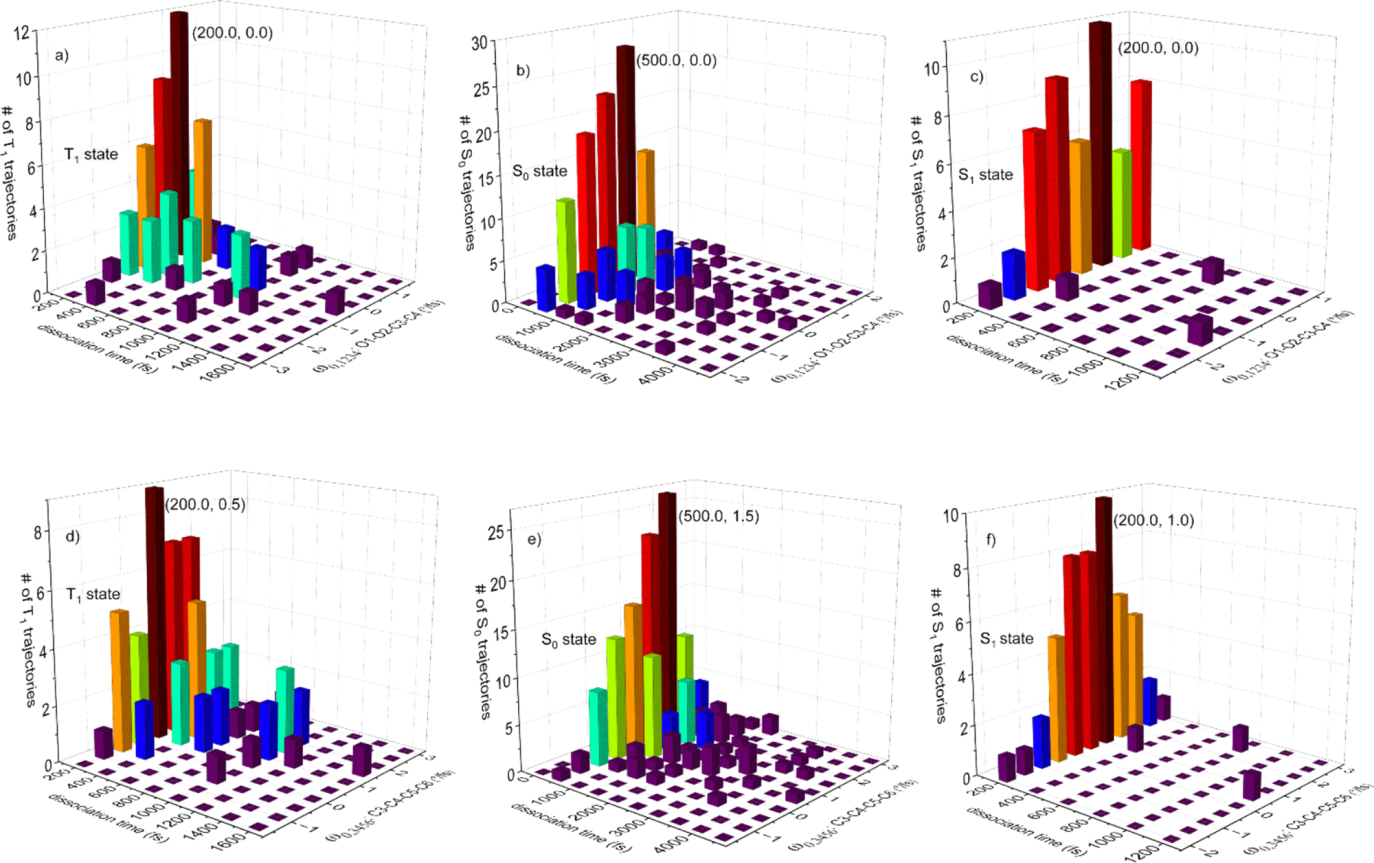
Distribution of the number of trajectories over the dissociation time (femtoseconds) and the initial angular velocity (degrees per femtosecond) of the O1–O2–C3–C4 dihedral angle for (a) state T_1_, (b) state S_0_, and (c) state S_1_. Distribution over the dissociation time and the initial angular velocity of the C3–C4–C5–C6 dihedral angle for (d) state T_1_, (e) state S_0_, and (f) state S_1_.

The ω_0,3456_ coordinates of the highest peaks of the T_1_, S_0_, and S_1_ trajectories are 0.5°/fs, 1.5°/fs, and 1.0°/fs, respectively; in other words, the most populated T_1_, S_0_, and S_1_ trajectories can be distinguished by the ω_0,3456_ coordinate (Figure 5d–f). In the subrange −2.0 ≤ ω_0,3456_ < −1.0 with a 20.0% RLSE, there is no T_1_ trajectory; thus, the trajectories with the ω_0,3456_ vicinal to the negative end tend to product states S_0_ and S_1_. Furthermore, Figure S5 shows that (1) the trajectories with the *v*_0,12_ (initial velocity of O1–O2) near the lower end (25.0% RLSE) incline to state S_0_, (2) the trajectories with the *v*_0,12_ adjacent to the upper end (12.5% RLSE) evolve least likely to state T_1_, (3) the trajectories with the *v*_0,34_ (initial velocity of C3–C4) vicinal to the negative end (23.1% RLSE) are apt to the singlet states, (4) the trajectories with the *v*_0,34_ close to the positive end (23.1% RLSE) tend to states T_1_ and S_0_, and (5) the trajectories with the *v*_0,56_ (initial velocity of C5–C6) neighboring to the positive end (33.3% RLSE) lean toward states S_0_ and S_1_. The initial dihedral angular velocities and/or initial velocities that take values near certain end points with considerable RLSE, e.g., the *v*_0,56_ adjacent to the positive end (33.3% RLSE), can be identified as the most characteristic features in trajectories, which incline to the given energy state, and their final energy states are “easiest” to classify.^47^

In conclusion, the thermal decomposition of *trans*-3,4-dimethyl-1,2-dioxetane has been studied by the TSH dynamic approach. The significant difference in the dissociation time between the long and short trajectory has been elucidated by the time spent in the triplet states and the number of frustrated dissociations, which can be regarded as the C–C bond oscillation between two adjacent local maxima of the potential energy of the active state. Adding the singlet excited states to the NAMD simulation postpones the dissociation by introducing several frustrated dissociations. However, inclusion of the triplet states with different *S*_*z*_ components results in more than a hundred frustrated dissociations, which delays the chemiluminescent decomposition remarkably. When the molecule ruptures its C–C bond through the excited state channel, e.g., T_1_ or S_1_, it has to pass over the T_1_ or S_1_ ridge to reach the corresponding energy product. The ground state ridge has not been found. The simulated quantum yield of state T_1_ matches the experimental result well. The dissociation half-times have been classified by the product energy states, and the order of the dissociation half-times is the same as that of the quantum yields for states T_1_, S_0_, and S_1_. This result provides evidence for the conjecture that the longer decomposition time leads to the higher quantum yield observed in the context of the mass effect of methylation. After the dissociation, the degeneracy of the ground and excited states has been lifted, and the label of the product energy state in terms of states T_1_, S_0_, and S_1_ has remained unchanged; thus, the ensemble-averaged populations of the MCH and diagonal states can reach equilibrium. Due to small energy gaps among states T_1,–1_, T_1,0_, and T_1,1_ (or T_2,–1_, T_2,0_, and T_2,1_), surface hops still exist even after the dissociation, and the populations of the triplets with different *S*_*z*_ components oscillate around certain values. The distribution of the trajectories over the initial velocities has revealed that the initial velocities near the end points with considerable relative sizes of the subranges make the product molecule tend to certain energy states, and these special initial velocities can be identified as the most characteristic features for classifying the trajectories as per their final energy states. Because of the stochastic nature of the TSH method, it would be interesting to check if there are fuzzy boundaries,^57^ which separate the trajectories by their product energy states, by applying a Bayesian support vector machine model.^58,59^ We will discuss these issues in the future.

## Supporting Information Available

Ground state, TS_O–O_, and TS_C–C_ structures of the TDMD and their coordinates optimized by the SA-CASSCF; comparison of the potential energy gaps among SA-CASSCF/6-31G, SA-CASSCF/6-31G*, and CASPT2/ANO-RCC-VDZP; time evolutions of the C3–C4 distance, O1–O2–C3–C4 dihedral angle, and potential energy of the active state for the S_0_ and S_1_ trajectories; nonadiabatic transitions between the MCH states for the S_0_ and S_1_ trajectories and for the ensemble; spin–orbital coupling matrix elements over spin components of spin-free eigenstates; and distributions of the trajectories with product states T_1_, S_0_, and S_1_ over the dissociation time and the initial velocity of O1–O2, C3–C4, and C5–C6, respectively (PDF)
