# Supplementary material for: Dissociation Time, Quantum Yield, and Dynamic Reaction Pathways in the Thermolysis of trans-3,4-Dimethyl-1,2-dioxetane
Source: J Phys Chem Lett. 2024 Feb 9;15(7):1846–55. doi: 10.1021/acs.jpclett.3c03578 (PMC10895692; doi:10.1021/acs.jpclett.3c03578)
Supplement: Supplementary file 1 — jz3c03578_si_001.pdf [file jz3c03578_si_001.pdf]

Supporting Information for

Dissociation Time, Quantum Yield and Dynamic Reaction Pathways in the Thermolysis of the Trans-3,4-dimethyl-1,2-dioxetane

Jian-Ge Zhou,[,+] Yinan Shu,[‡] Yuchen Wang,[&] Jerzy Leszczynski,[*,+] Oleg Prezhdo[*,$]*

[+]*Interdisciplinary Nanotoxicity Center, Department of Chemistry, Physics and Atmospheric Sciences, Jackson State University, Jackson, Mississippi 39217, United States*

[‡]*Department of Chemistry and Supercomputing Institute, University of Minnesota, Minneapolis, Minnesota 55455-0431, United States*

[&]*Department of Chemistry and the James Franck Institute, The University of Chicago, Chicago, Illinois 60637, United States*

[$]*Department of Chemistry and Department of Physics and Astronomy, University of Southern California, Los Angeles, CA 90089, United States*

*E-mail: jiange.zhou@jsums.edu; jerzy@icnanotox.org and prezhdo@usc.edu.

1. **The structures of the O-O transition state (TS_{O-O}) and C-C transition state (TS_{C-C}) of the trans-3,4-dimethyl-1,2-dioxetane, and the corresponding coordinates optimized by the SA8--CASSCF(12e,10o)/6-31G**

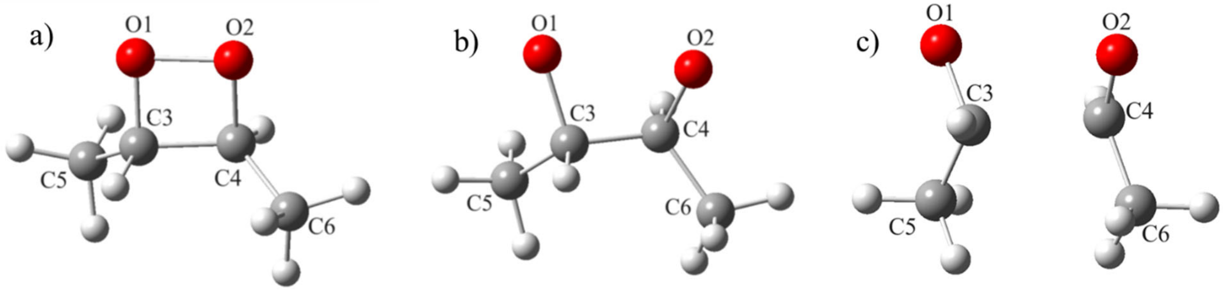

Figure S1 The structures of trans-3,4-dimethyl-1,2-dioxetane, a) the ground state of the molecule, b) the O-O transition state, c) the C-C transition state.

A) The coordinates (Å) of the TS_{O-O} state on the S_0 PES optimized at SA8-CASSCF(12e,10o)/6-31G

O	-1.31261675	0.67512797	-0.52303875
O	1.04443270	0.90981818	0.13476240
C	-0.81852255	-0.54219490	0.17467070
C	0.66374236	-0.51211749	-0.12009682
C	-1.61496311	-1.69577383	-0.39841033
C	1.52926770	-1.36745536	0.78922085
H	0.84658644	-0.72185559	-1.16343211
H	-1.37899783	-2.61228615	0.12708375
H	-0.97768895	-0.47971953	1.25063309
H	-1.40128921	-1.82554415	-1.45100993
H	-2.67614099	-1.51146957	-0.28620784
H	1.27937666	-2.41550061	0.67416479
H	2.57600796	-1.24541943	0.54327493
H	1.38926458	-1.09156855	1.82672728

B) The coordinates (Å) of the TS_{C-C} state on the T_1 PES optimized at SA8-CASSCF(12e,10o)/6-31G

O	-1.59040711	0.60149734	-0.58345906
O	1.05689164	0.95808212	0.89293587
C	-1.24468169	-0.46982874	0.10647084
C	0.82140341	-0.21715068	0.11099934
C	-1.48383513	-1.82607618	-0.50860173
C	1.50581109	-1.41850732	0.71440958
H	1.00548889	-0.00751798	-0.92768655
H	-1.02149547	-2.61688653	0.06578135
H	-1.29674668	-0.38980446	1.17981008
H	-1.11039026	-1.85889281	-1.52443614
H	-2.54909013	-2.02648458	-0.54406768
H	1.12729296	-1.63082284	1.70661813
H	1.35544989	-2.29136805	0.09598716
H	2.57276760	-1.24219826	0.79358082

C) The coordinates (Å) of the TS_{C-C} state on the S_1 PES optimized at SA8-CASSCF(12e,10o)/6-31G

O	-1.68683492	0.60051400	-0.61776339
O	0.95145298	0.93716777	0.89109363
C	-1.15243110	-0.51193151	0.14364639
C	0.94253815	-0.15366689	0.15751462
C	-1.47796215	-1.82620489	-0.51401686
C	1.55390787	-1.41293131	0.71376468
H	0.93768681	-0.02194120	-0.91183936
H	-1.11041413	-2.65118941	0.07951431
H	-1.38902388	-0.38852005	1.18474089

H	-1.03950767	-1.89241599	-1.50235391
H	-2.55185681	-1.93841855	-0.61799491
H	1.23932127	-1.57161058	1.73728828
H	1.29654104	-2.28114809	0.12304463
H	2.63504156	-1.32366229	0.71170299

2. The comparison of the potential energy gaps among SA8-CASSCF(12e,10o)/6-31G, SA8-CASSCF(12e,10o)/6-31G* and CASPT2(12e,10o)/ANO-RCC-VDZP approach

The potential energy gaps between the excited and ground state calculated by the SA8-CASSCF(12e,10o)/6-31G, SA8-CASSCF(12e,10o)/6-31G* and CASPT2(12e,10o)/ANO-RCC-VDZP methods are compared. The results obtained by the CASSCF/6-31G match that of the

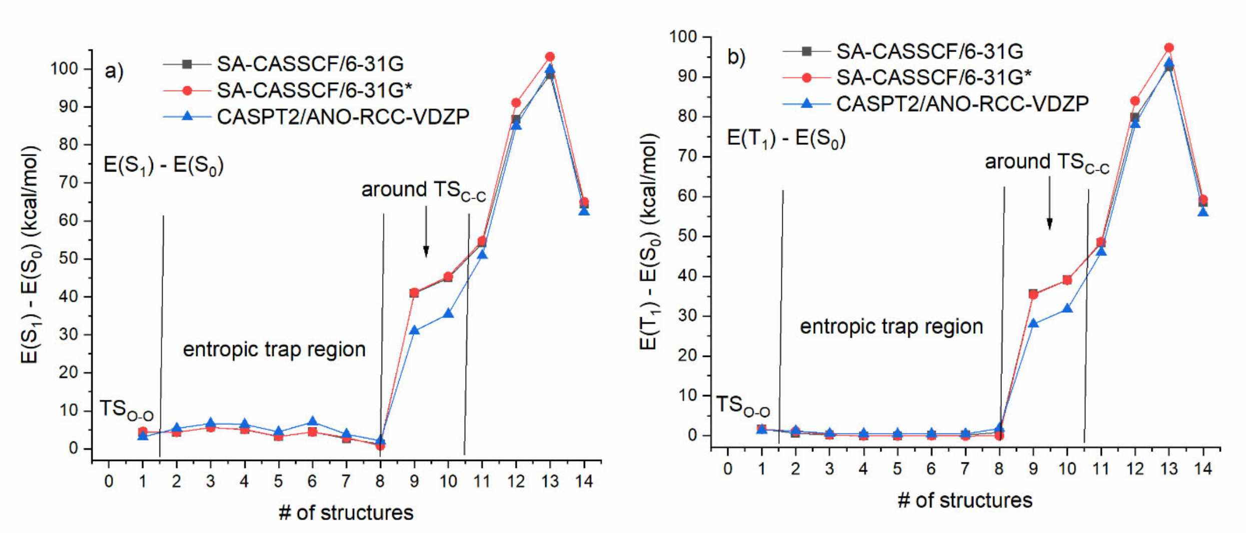

Figure S2 The potential energy gaps (kcal/mol) between excited and ground state at the 14 configurations along the short S_0 trajectory (traj. 84) are compared among SA-CASSCF/6-31G, SA-CASSCF/6-31G* and CASPT2/ANO-RCC-VDZP: a) $E(S_1)$-$E(S_0)$, b) $E(T_1)$-$E(S_0)$.

CASSCF/6-31G*. In the $TS_{O\text{-}O}$, biradical (i.e., the entropic trap) and product region, the energy gap errors between SA-CASSCF/6-31G and CASPT2/ANO-RCC-VDZP are around 3 kcal/mol, as show in Figure S2. Around $TS_{C\text{-}C}$, the gap errors between the SA-CASSCF/6-31G and CASPT2/ANO-RCC-VDZP are around 10 kcal/mol (we assume that CASPT2/ANO-RCC-VDZP can provide accurate results). This is attributed to that SA-CASSCF overestimates the energy barrier of the $TS_{C\text{-}C}$ than CASPT2. The energy difference of E(T_1 at $TS_{C\text{-}C}$) - E(S_0 at $TS_{O\text{-}O}$) computed via SA-CASSCF/6-31G is higher than that evaluated via CASPT2/ANO-RCC-

VDZP by 14.1 kcal/mol (0.61eV). To make the trajectories that pass over the energy barrier of the $TS_{C\text{-}C}$ calculated by CASPT2 also overcome the activation energy evaluated by SA-CASSCF, we add 0.61eV to the initial kinetic energy of the $TS_{O\text{-}O}$ as the compensation for the SA-CASSCF approach. Along the O-O reaction coordinate, the extra initial velocities has been added to make all completed trajectories dissociate. At structure 12 and 13, the energy differences computed via CASSCF(12e,10o)/6-31G are closer to those of CASPT2/ANO-RCC-VDZP than via SA8-CASSCF(12e,10o)/6-31G* because of the error offset from CASSCF and 6-31G.

3. **The time evolutions of the distance between C3 and C4, dihedral angle O1-O2-C3-C4, and the potential energy of the active state**

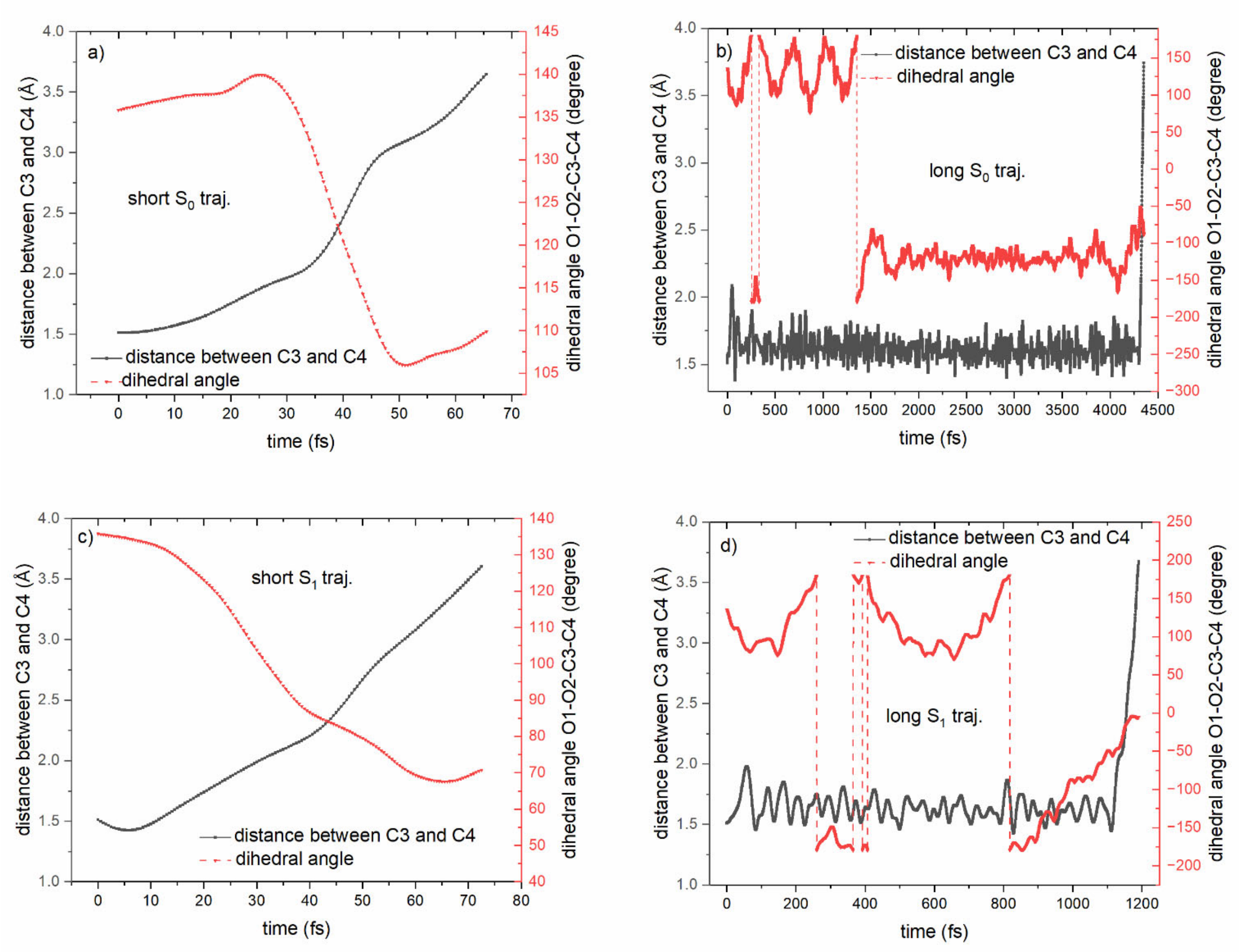

Figure S3 The time evolutions of the C3-C4 distance (Å) and the dihedral angle O1-O2-C3-C4 (degree) for a) S_0 short trajectory, b) S_0 long trajectory, c) S_1 short trajectory, d) S_1 long trajectory.

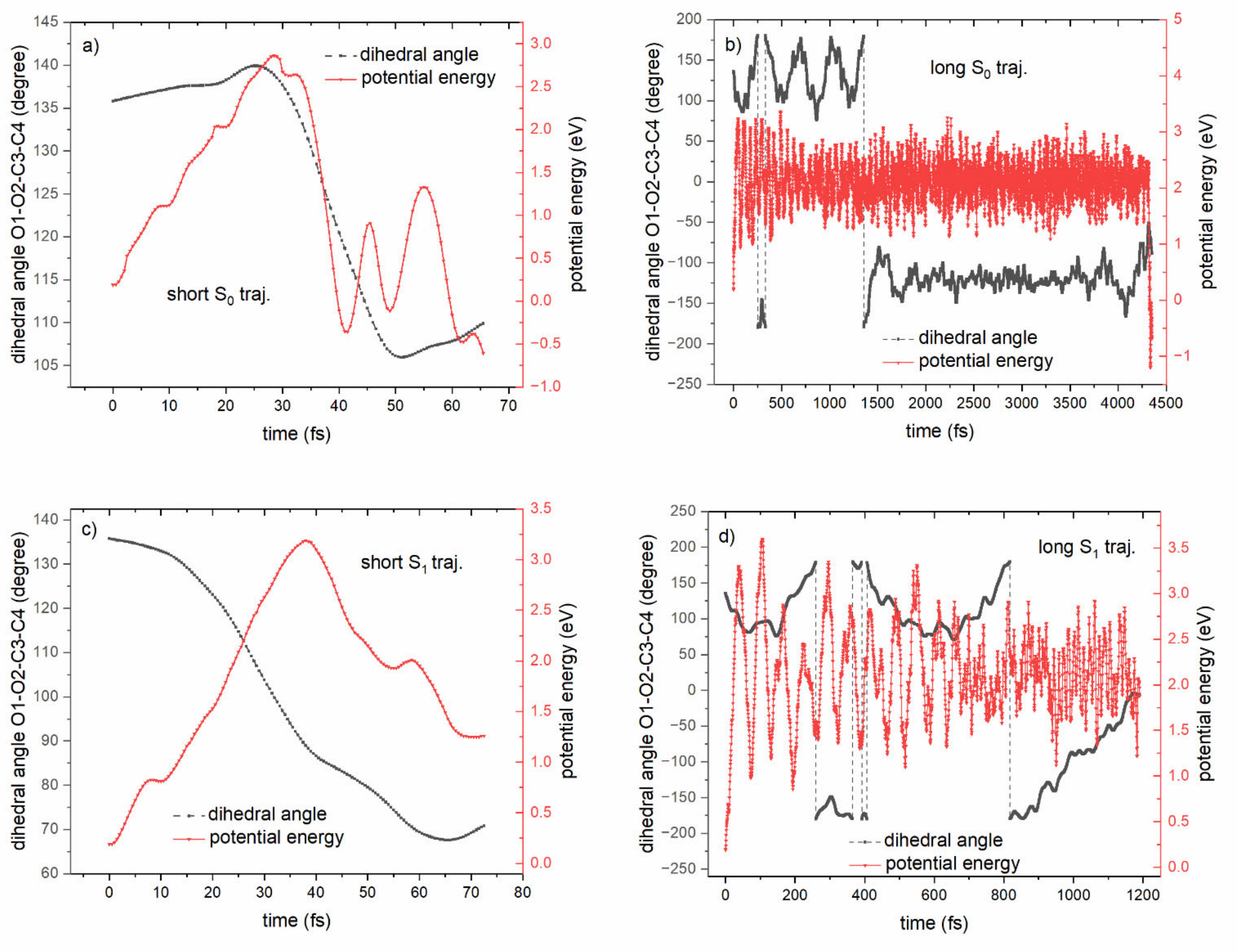

Figure S4 The time evolutions of the dihedral angle O1-O2-C3-C4 (degree) and the potential energy (eV) of the active state for a) S_0 short trajectory, b) S_0 long trajectory, c) S_1 short trajectory, d) S_1 long trajectory.

4. The nonadiabatic transitions between the MCH states in the four trajectories and ensemble

Let's look at the steps in a toy trajectory listed in the following column:
Step# MCH state

0. S_0
1. S_0
2. S_0

----

3. S_2
4. S_2
5. S_2
6. S_2
7. S_2

----

8. S_1
9. S_1
10. S_1
11. S_1

The above steps are rewritten in the following table:

The number of the nonadiabatic transitions between the MCH states in a toy trajectory

	S_0	S_1	S_2
S_0	2	0	1
S_1	0	3	0
S_2	0	1	4

Since the starting state is the S_0 state (i.e., $TS_{O\text{-}O}$ in the S_0), the steps in the S_0, S_1 and S_2, as shown in the above column, are 2, 4 and 5 respectively. The above table can be regarded as a 3 × 3 matrix, and its matrix elements are denoted as a(i,j). Look at the a(3,3), i.e., a(S_2,S_2) =4. The diagonal element a(S_2,S_2) represents the number of transitions within the S_2 state, that is, from step3 to step4, …, from step6 to step7, totally four transitions. The off-diagonal element a(S_0,S_2), i.e., a(1,3)=1, represents the transition from S_0 to S_2, that is, from step2 to step3 in the toy trajectory. The number of the steps on the S_2 is a(S_2,S_2) + all the off-diagonal elements along column 3 in the table, that is, 4+1+0=5, which is the number of the total steps on the S_2 state.

Table S1 The number of the nonadiabatic transitions between the MCH states in the S_0 short trajectory.

	S_0	S_1	S_2	S_3	$T_{1,-1}$	$T_{2,-1}$	$T_{1,0}$	$T_{2,0}$	$T_{1,1}$	$T_{2,1}$
S_0	82	0	1	0	0	0	0	0	0	0
S_1	1	27	0	0	0	0	0	0	0	1
S_2	0	0	1	1	0	0	0	0	0	0
S_3	0	1	0	21	0	0	0	0	0	0
$T_{1,-1}$	0	0	0	0	0	0	0	0	0	0
$T_{2,-1}$	0	1	0	0	0	0	0	0	0	0
$T_{1,0}$	0	0	0	0	0	0	0	0	0	0
$T_{2,0}$	0	0	0	0	0	0	0	0	0	0
$T_{1,1}$	0	0	0	0	0	0	0	0	0	0
$T_{2,1}$	0	0	0	0	0	1	0	0	0	0

Table S2 The number of the nonadiabatic transitions between the MCH states in the S_0 long trajectory.

	S_0	S_1	S_2	S_3	$T_{1,-1}$	$T_{2,-1}$	$T_{1,0}$	$T_{2,0}$	$T_{1,1}$	$T_{2,1}$
S_0	796	20	0	1	19	0	24	1	20	2
S_1	18	531	6	2	0	16	10	20	7	15
S_2	2	12	207	2	0	2	2	2	0	7
S_3	0	0	7	136	0	0	1	0	0	0
$T_{1,-1}$	18	3	1	0	788	6	68	6	41	2
$T_{2,-1}$	1	8	8	1	3	686	5	47	3	27
$T_{1,0}$	24	12	0	0	80	2	1796	24	73	2
$T_{2,0}$	1	22	3	0	4	43	25	1207	7	48
$T_{1,1}$	23	4	1	0	36	4	78	4	906	3
$T_{2,1}$	0	13	3	2	3	30	4	49	2	557

Table S3 The number of the nonadiabatic transitions between the MCH states in the S_1 short trajectory.

	S_0	S_1	S_2	S_3	$T_{1,-1}$	$T_{2,-1}$	$T_{1,0}$	$T_{2,0}$	$T_{1,1}$	$T_{2,1}$
S_0	53	0	0	0	1	0	0	0	0	0
S_1	1	87	0	0	0	0	0	0	0	0
S_2	0	0	0	0	0	0	0	0	0	0
S_3	0	0	0	0	0	0	0	0	0	0
$T_{1,-1}$	1	0	0	0	2	0	0	0	0	0
$T_{2,-1}$	0	0	0	0	0	0	0	0	0	0
$T_{1,0}$	0	0	0	0	0	0	0	0	0	0
$T_{2,0}$	0	0	0	0	0	0	0	0	0	0
$T_{1,1}$	0	0	0	0	0	0	0	0	0	0
$T_{2,1}$	0	0	0	0	0	0	0	0	0	0

Table S4 The number of the nonadiabatic transitions between the MCH states in the S_1 long trajectory.

	S_0	S_1	S_2	S_3	$T_{1,-1}$	$T_{2,-1}$	$T_{1,0}$	$T_{2,0}$	$T_{1,1}$	$T_{2,1}$
S_0	368	5	0	0	3	2	9	0	3	0
S_1	7	325	6	3	0	2	1	9	1	8
S_2	0	6	69	2	0	1	0	1	0	1
S_3	1	0	4	138	0	0	0	0	0	0
$T_{1,-1}$	4	0	0	0	93	2	11	2	8	1
$T_{2,-1}$	0	8	0	0	0	58	1	8	1	2
$T_{1,0}$	5	4	0	0	18	1	599	4	12	2
$T_{2,0}$	2	9	0	0	2	10	8	252	1	8
$T_{1,1}$	4	2	0	0	4	0	16	0	98	0
$T_{2,1}$	0	2	1	0	1	2	0	16	0	124

Table S5 The number of the nonadiabatic transitions between the MCH states in the ensemble of all the trajectories.

	S_0	S_1	S_2	S_3	$T_{1,-1}$	$T_{2,-1}$	$T_{1,0}$	$T_{2,0}$	$T_{1,1}$	$T_{2,1}$
S_0	64403	551	65	8	493	34	880	73	391	42
S_1	583	32592	474	75	52	496	138	597	119	379
S_2	115	445	14558	266	16	143	13	152	10	118
S_3	8	74	272	9870	2	13	3	13	2	9
$T_{1,-1}$	495	57	8	3	30817	168	1675	117	1299	102
$T_{2,-1}$	55	495	147	13	180	20816	105	1333	115	995
$T_{1,0}$	853	156	12	5	1703	117	39993	408	1431	89
$T_{2,0}$	82	588	174	12	105	1297	402	30254	85	1158
$T_{1,1}$	418	133	6	0	1257	134	1444	86	25946	160
$T_{2,1}$	50	362	120	14	103	1036	83	1124	160	18040

5. The spin-orbital (SO) coupling matrix elements over spin components of spin-free eigenstates

Table S6 The SO coupling matrix elements (cm^{-1}) over spin components of spin-free eigenstates via SA-CASSCF/6-31G for $TS_{O\text{-}O}$: (Print threshold: 10.000 cm^{-1})

I1	S1	MS1	I2	S2	MS2	Real part	Imag part	Absolute
5	1	-1	2	0	0	34.462	-7.561	35.282
5	1	-1	3	0	0	-23.631	-10.659	25.924
5	1	-1	4	0	0	3.43	-9.936	10.511
6	1	0	1	0	0	0	14.629	14.629
6	1	0	3	0	0	0	-23.598	23.598
6	1	0	4	0	0	0	-17.114	17.114
7	1	1	2	0	0	34.462	7.561	35.282
7	1	1	3	0	0	-23.631	10.659	25.924
7	1	1	4	0	0	3.43	9.936	10.511
8	1	-1	1	0	0	-38.038	8.762	39.034
8	1	-1	3	0	0	7.456	7.369	10.483
8	1	-1	4	0	0	-29.9	-10.515	31.695
8	1	-1	6	1	0	-41.223	7.691	41.935
9	1	0	3	0	0	0	14.597	14.597
9	1	0	4	0	0	0	-24.34	24.34
9	1	0	5	1	-1	41.223	7.691	41.935
9	1	0	7	1	1	-41.223	7.691	41.935
10	1	1	1	0	0	-38.038	-8.762	39.034
10	1	1	3	0	0	7.456	-7.369	10.483
10	1	1	4	0	0	-29.9	10.515	31.695
10	1	1	6	1	0	41.223	7.691	41.935
11	1	-1	1	0	0	28.154	10.205	29.946
11	1	-1	2	0	0	-29.111	-0.447	29.114
11	1	-1	3	0	0	18.46	2.218	18.592
11	1	-1	4	0	0	34.727	-8.254	35.694
11	1	-1	5	1	-1	0	22.259	22.259
11	1	-1	6	1	0	-22.571	-9.846	24.625
11	1	-1	8	1	-1	0	-20.61	20.61
11	1	-1	9	1	0	9.125	10.447	13.871
12	1	0	1	0	0	0	24.129	24.129
12	1	0	5	1	-1	22.571	-9.846	24.625
12	1	0	7	1	1	-22.571	-9.846	24.625
12	1	0	8	1	-1	-9.125	10.447	13.871
12	1	0	10	1	1	9.125	10.447	13.871
13	1	1	1	0	0	28.154	-10.205	29.946
13	1	1	2	0	0	-29.111	0.447	29.114
13	1	1	3	0	0	18.46	-2.218	18.592

13	1	1	4	0	0	34.727	8.254	35.694
13	1	1	6	1	0	22.571	-9.846	24.625
13	1	1	7	1	1	0	-22.259	22.259
13	1	1	9	1	0	-9.125	10.447	13.871
13	1	1	10	1	1	0	20.61	20.61
14	1	-1	1	0	0	16.854	2.533	17.043
14	1	-1	2	0	0	28.147	11.428	30.378
14	1	-1	3	0	0	-32.272	11.722	34.335
14	1	-1	4	0	0	14.124	0.189	14.125
14	1	-1	5	1	-1	0	14.304	14.304
14	1	-1	6	1	0	-29.331	-4.763	29.716
14	1	-1	8	1	-1	0	21.766	21.766
14	1	-1	9	1	0	-25.312	-9.481	27.03
14	1	-1	11	1	-1	0	-15.191	15.191
14	1	-1	12	1	0	-32.066	11.59	34.097
15	1	0	2	0	0	0	26.005	26.005
15	1	0	3	0	0	0	15.221	15.221
15	1	0	5	1	-1	29.331	-4.763	29.716
15	1	0	7	1	1	-29.331	-4.763	29.716
15	1	0	8	1	-1	25.312	-9.481	27.03
15	1	0	10	1	1	-25.312	-9.481	27.03
15	1	0	11	1	-1	32.066	11.59	34.097
15	1	0	13	1	1	-32.066	11.59	34.097
16	1	1	1	0	0	16.854	-2.533	17.043
16	1	1	2	0	0	28.147	-11.428	30.378
16	1	1	3	0	0	-32.272	-11.722	34.335
16	1	1	4	0	0	14.124	-0.189	14.125
16	1	1	6	1	0	29.331	-4.763	29.716
16	1	1	7	1	1	0	-14.304	14.304
16	1	1	9	1	0	25.312	-9.481	27.03
16	1	1	10	1	1	0	-21.766	21.766
16	1	1	12	1	0	32.066	11.59	34.097
16	1	1	13	1	1	0	15.191	15.191

Table S7 The SO coupling matrix elements (cm^{-1}) over spin components of spin-free eigenstates via SA-CASSCF/6-31G* for TS_{O-O}: (Print threshold: 10.000 cm^{-1})

I1	S1	MS1	I2	S2	MS2	Real part	Imag part	Absolute
5	1	-1	1	0	0	8.01	-6.239	10.153
5	1	-1	2	0	0	-31.312	-10.513	33.03
5	1	-1	3	0	0	27.938	-11.067	30.05
6	1	0	2	0	0	0	-24.609	24.609
6	1	0	3	0	0	0	-14.615	14.615
6	1	0	4	0	0	0	-13.909	13.909
7	1	1	1	0	0	8.01	6.239	10.153
7	1	1	2	0	0	-31.312	10.513	33.03
7	1	1	3	0	0	27.938	11.067	30.05
8	1	-1	1	0	0	30.993	12.865	33.557
8	1	-1	4	0	0	-34.667	9.898	36.052
8	1	-1	5	1	-1	0	-25.668	25.668
8	1	-1	6	1	0	36.637	10.491	38.11
9	1	0	1	0	0	0	28.471	28.471
9	1	0	4	0	0	0	11.193	11.193
9	1	0	5	1	-1	-36.637	10.491	38.11
9	1	0	7	1	1	36.637	10.491	38.11
10	1	1	1	0	0	30.993	-12.865	33.557
10	1	1	4	0	0	-34.667	-9.898	36.052
10	1	1	6	1	0	-36.637	10.491	38.11
10	1	1	7	1	1	0	25.668	25.668
11	1	-1	1	0	0	-34.956	7.404	35.732
11	1	-1	2	0	0	-26.955	-1.645	27.005
11	1	-1	3	0	0	17.894	-0.086	17.894
11	1	-1	4	0	0	30.444	10.682	32.264
11	1	-1	5	1	-1	0	11.356	11.356
11	1	-1	6	1	0	29.307	-9.207	30.719
11	1	-1	8	1	-1	0	13.421	13.421
11	1	-1	9	1	0	8.664	-8.337	12.024
12	1	0	4	0	0	0	24.524	24.524
12	1	0	5	1	-1	-29.307	-9.207	30.719
12	1	0	7	1	1	29.307	-9.207	30.719
12	1	0	8	1	-1	-8.664	-8.337	12.024
12	1	0	10	1	1	8.664	-8.337	12.024
13	1	1	1	0	0	-34.956	-7.404	35.732
13	1	1	2	0	0	-26.955	1.645	27.005
13	1	1	3	0	0	17.894	0.086	17.894
13	1	1	4	0	0	30.444	-10.682	32.264
13	1	1	6	1	0	-29.307	-9.207	30.719

13	1	1	7	1	1	0	-11.356	11.356
13	1	1	9	1	0	-8.664	-8.337	12.024
13	1	1	10	1	1	0	-13.421	13.421
14	1	-1	1	0	0	14.907	-1.669	15
14	1	-1	2	0	0	-36.823	7.877	37.657
14	1	-1	3	0	0	23.87	14.275	27.812
14	1	-1	4	0	0	-12.513	-2.387	12.739
14	1	-1	6	1	0	-26.764	0.649	26.771
14	1	-1	9	1	0	34.044	-7.571	34.875
14	1	-1	11	1	-1	0	-29.271	29.271
14	1	-1	12	1	0	24.217	13.844	27.895
15	1	0	3	0	0	0	29.636	29.636
15	1	0	5	1	-1	26.764	0.649	26.771
15	1	0	7	1	1	-26.764	0.649	26.771
15	1	0	8	1	-1	-34.044	-7.571	34.875
15	1	0	10	1	1	34.044	-7.571	34.875
15	1	0	11	1	-1	-24.217	13.844	27.895
15	1	0	13	1	1	24.217	13.844	27.895
16	1	1	1	0	0	14.907	1.669	15
16	1	1	2	0	0	-36.823	-7.877	37.657
16	1	1	3	0	0	23.87	-14.275	27.812
16	1	1	4	0	0	-12.513	2.387	12.739
16	1	1	6	1	0	26.764	0.649	26.771
16	1	1	9	1	0	-34.044	-7.571	34.875
16	1	1	12	1	0	-24.217	13.844	27.895
16	1	1	13	1	1	0	29.271	29.271

Table S8 The SO coupling matrix elements (cm^{-1}) over spin components of spin-free eigenstates via SA-CASSCF/ANO-RCC-VDZP for TS_{O-O}: (Print threshold: 10.000 cm^{-1})

I1	S1	MS1	I2	S2	MS2	Real part	Imag part	Absolute
5	1	-1	1	0	0	9.593	-10.253	14.041
5	1	-1	2	0	0	-42.963	5.993	43.378
5	1	-1	3	0	0	13.306	13.846	19.203
5	1	-1	4	0	0	1.282	14.386	14.443
6	1	0	1	0	0	0	-15.067	15.067
6	1	0	3	0	0	0	27.303	27.303
6	1	0	4	0	0	0	25.564	25.564
7	1	1	1	0	0	9.593	10.253	14.041
7	1	1	2	0	0	-42.963	-5.993	43.378
7	1	1	3	0	0	13.306	-13.846	19.203
7	1	1	4	0	0	1.282	-14.386	14.443
8	1	-1	1	0	0	-15.885	14.993	21.843
8	1	-1	2	0	0	-38.494	3.152	38.622
8	1	-1	3	0	0	16.69	9.953	19.432
8	1	-1	4	0	0	-2.52	-14.632	14.847
8	1	-1	6	1	0	55.775	-2.275	55.822
9	1	0	1	0	0	0	24.057	24.057
9	1	0	3	0	0	0	20.413	20.413
9	1	0	4	0	0	0	-26.324	26.324
9	1	0	5	1	-1	-55.775	-2.275	55.822
9	1	0	7	1	1	55.775	-2.275	55.822
10	1	1	1	0	0	-15.885	-14.993	21.843
10	1	1	2	0	0	-38.494	-3.152	38.622
10	1	1	3	0	0	16.69	-9.953	19.432
10	1	1	4	0	0	-2.52	14.632	14.847
10	1	1	6	1	0	-55.775	-2.275	55.822
11	1	-1	1	0	0	58.353	5.128	58.578
11	1	-1	4	0	0	55.67	-2.481	55.725
11	1	-1	5	1	-1	0	-26.193	26.193
11	1	-1	6	1	0	2.584	14.17	14.404
11	1	-1	8	1	-1	0	-25.141	25.141
11	1	-1	9	1	0	1.008	14.022	14.058
12	1	0	1	0	0	0	20.134	20.134
12	1	0	5	1	-1	-2.584	14.17	14.404
12	1	0	7	1	1	2.584	14.17	14.404
12	1	0	8	1	-1	-1.008	14.022	14.058
12	1	0	10	1	1	1.008	14.022	14.058
13	1	1	1	0	0	58.353	-5.128	58.578
13	1	1	4	0	0	55.67	2.481	55.725

13	1	1	6	1	0	-2.584	14.17	14.404
13	1	1	7	1	1	0	26.193	26.193
13	1	1	9	1	0	-1.008	14.022	14.058
13	1	1	10	1	1	0	25.141	25.141
14	1	-1	2	0	0	46.44	10.145	47.536
14	1	-1	3	0	0	-31.934	17.4	36.367
14	1	-1	5	1	-1	0	-13.621	13.621
14	1	-1	6	1	0	41.072	3.228	41.199
14	1	-1	8	1	-1	0	14.99	14.99
14	1	-1	9	1	0	-42.316	-3.821	42.488
14	1	-1	11	1	-1	0	-28.179	28.179
14	1	-1	12	1	0	-18.497	17.751	25.637
15	1	0	2	0	0	0	26.934	26.934
15	1	0	3	0	0	0	24.745	24.745
15	1	0	5	1	-1	-41.072	3.228	41.199
15	1	0	7	1	1	41.072	3.228	41.199
15	1	0	8	1	-1	42.316	-3.821	42.488
15	1	0	10	1	1	-42.316	-3.821	42.488
15	1	0	11	1	-1	18.497	17.751	25.637
15	1	0	13	1	1	-18.497	17.751	25.637
16	1	1	2	0	0	46.44	-10.145	47.536
16	1	1	3	0	0	-31.934	-17.4	36.367
16	1	1	6	1	0	-41.072	3.228	41.199
16	1	1	7	1	1	0	13.621	13.621
16	1	1	9	1	0	42.316	-3.821	42.488
16	1	1	10	1	1	0	-14.99	14.99
16	1	1	12	1	0	18.497	17.751	25.637
16	1	1	13	1	1	0	28.179	28.179

Table S9 The SO coupling matrix elements (cm^{-1}) over spin components of spin-free eigenstates via CASPT2/ANO-RCC-VDZP for $TS_{O\text{-}O}$: (Print threshold: 10.000 cm^{-1})

I1	S1	MS1	I2	S2	MS2	Real part	Imag part	Absolute
5	1	-1	2	0	0	-64.201	4.477	64.357
5	1	-1	3	0	0	21.731	14.287	26.007
6	1	0	3	0	0	0	29.496	29.496
7	1	1	2	0	0	-64.201	-4.477	64.357
7	1	1	3	0	0	21.731	-14.287	26.007
8	1	-1	1	0	0	-1.138	18.231	18.267
8	1	-1	2	0	0	-10.757	-1.923	10.927
8	1	-1	4	0	0	13.671	-20.391	24.55
8	1	-1	5	1	-1	0	-14.651	14.651
8	1	-1	6	1	0	44.337	3.609	44.484
9	1	0	1	0	0	0	31.809	31.809
9	1	0	4	0	0	0	-33.372	33.372
9	1	0	5	1	-1	-44.337	3.609	44.484
9	1	0	7	1	1	44.337	3.609	44.484
10	1	1	1	0	0	-1.138	-18.231	18.267
10	1	1	2	0	0	-10.757	1.923	10.927
10	1	1	4	0	0	13.671	20.391	24.55
10	1	1	6	1	0	-44.337	3.609	44.484
10	1	1	7	1	1	0	14.651	14.651
11	1	-1	1	0	0	62.557	-0.134	62.557
11	1	-1	3	0	0	-12.13	0.286	12.133
11	1	-1	4	0	0	51.988	3.593	52.112
11	1	-1	5	1	-1	0	-36.964	36.964
11	1	-1	6	1	0	-13.102	21.898	25.518
12	1	0	1	0	0	0	11.762	11.762
12	1	0	4	0	0	0	16.319	16.319
12	1	0	5	1	-1	13.102	21.898	25.518
12	1	0	7	1	1	-13.102	21.898	25.518
13	1	1	1	0	0	62.557	0.134	62.557
13	1	1	3	0	0	-12.13	-0.286	12.133
13	1	1	4	0	0	51.988	-3.593	52.112
13	1	1	6	1	0	13.102	21.898	25.518
13	1	1	7	1	1	0	36.964	36.964
14	1	-1	2	0	0	38.948	9.803	40.163
14	1	-1	3	0	0	-26.025	20.393	33.063
14	1	-1	6	1	0	11.916	-0.623	11.933
14	1	-1	8	1	-1	0	13.543	13.543
14	1	-1	9	1	0	-68.101	-0.287	68.102
14	1	-1	11	1	-1	0	-27.575	27.575

14	1	-1	12	1	0	1.427	15.296	15.363
15	1	0	2	0	0	0	24.9	24.9
15	1	0	3	0	0	0	31.16	31.16
15	1	0	5	1	-1	-11.916	-0.623	11.933
15	1	0	7	1	1	11.916	-0.623	11.933
15	1	0	8	1	-1	68.101	-0.287	68.102
15	1	0	10	1	1	-68.101	-0.287	68.102
15	1	0	11	1	-1	-1.427	15.296	15.363
15	1	0	13	1	1	1.427	15.296	15.363
16	1	1	2	0	0	38.948	-9.803	40.163
16	1	1	3	0	0	-26.025	-20.393	33.063
16	1	1	6	1	0	-11.916	-0.623	11.933
16	1	1	9	1	0	68.101	-0.287	68.102
16	1	1	10	1	1	0	-13.543	13.543
16	1	1	12	1	0	-1.427	15.296	15.363
16	1	1	13	1	1	0	27.575	27.575

The S-T mixing between the singlet and triplet states is responsible for triplet quantum yield, phosphorescence intensity and lifetime, and the T-T mixing between two triplet states with different z-components of the spin provides anisotropic deviation of the g-factor in EPR spectra and of the Zeeman energy in an external magnetic field. The S-T mixing is larger if the numerator $\langle S_m|H_{SO}|T_n\rangle$ is larger or the denominator $E(T_n)-E(S_m)$ is smaller. Since the potential energy order at TS_{O-O} is $S_0 < T_1 < S_1 < T_2 < T_3 < S_2 < T_4 < S_3$, the large absolute SO matrix elements between the singlet and triplet states with adjacent energy levels (e.g., $S_1 \rightarrow T_1$, $S_1 \rightarrow T_2$ instead of $S_1 \rightarrow T_3$) play the dominant role in the triplet quantum yield. Comparing Table S6 with Table S9, the S-T mixing of SA-CASSCF/6-31G was calibrated by that of CASPT2/ANO-RCC-VDZP. Furthermore, the SO matrix elements of SA-CASSCF/6-31G are closer to those of CASPT2/ANO-RCC-VDZP than SA-CASSCF/6-31G*, which implies that the basis set 6-31G performs better than 6-31G* at the SA-CASSCF level.

6. The distribution of the number of trajectories over the dissociation time and the initial velocities, and the distribution of the trajectories over the two different initial velocities

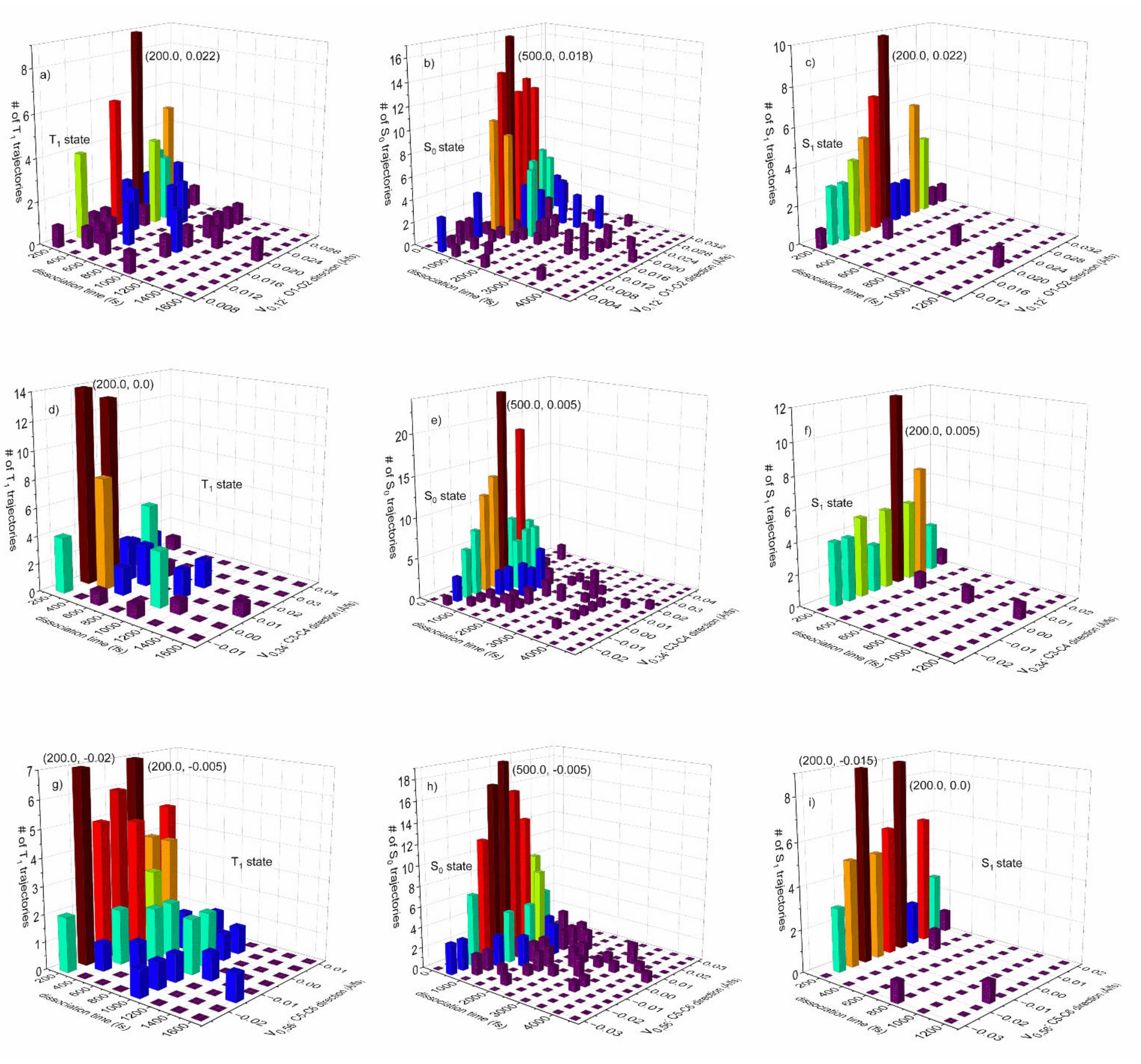

Figure S5 The distribution of the number of trajectories over the dissociation time (fs) and the initial velocities (Å/fs). Besides over the dissociation time, it's also over the initial velocity of the O1-O2 for a) T_1 state, b) S_0 state, c) S_1 state; over the initial velocity of the C3-C4 for d) T_1 state, e) S_0 state, f) S_1 state; and over the initial velocity of the C5-C6 for g) T_1 state, h) S_0 state, i) S_1 state.

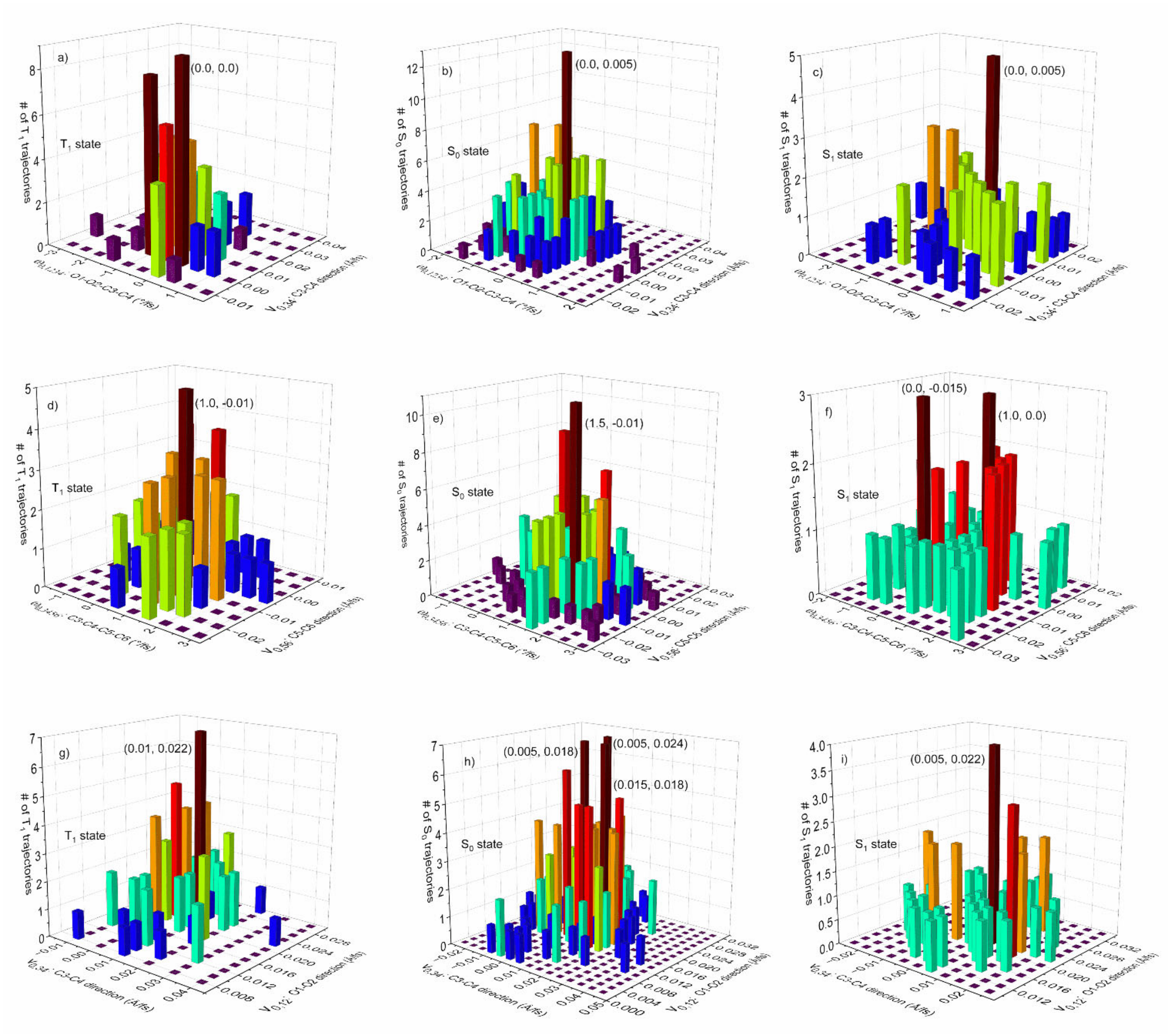

Figure S6 The distribution of the number of trajectories over two different initial velocitiee. Over the initial velocities of the O1-O2-C3-C4 (degree/fs) and C3-C4 (Å/fs) for a) T_1 state, b) S_0 state, c) S_1 state; over the initial velocities of the C3-C4-C5-C6 (degree/fs) and C5-C6 (Å/fs) for d) T_1 state, e) S_0 state, f) S_1 state; and over the initial velocities of the C3-C4 (Å/fs) and O1-O2 (Å/fs) for g) T_1 state, h) S_0 state, i) S_1 state.
